# Multi-modal investigation of the bone micro- and ultrastructure, and elemental distribution in the presence of Mg-xGd screws at mid-term healing stages

**DOI:** 10.1016/j.bioactmat.2024.07.019

**Published:** 2024-09-03

**Authors:** Kamila Iskhakova, Hanna Cwieka, Svenja Meers, Heike Helmholz, Anton Davydok, Malte Storm, Ivo Matteo Baltruschat, Silvia Galli, Daniel Pröfrock, Olga Will, Mirko Gerle, Timo Damm, Sandra Sefa, Weilue He, Keith MacRenaris, Malte Soujon, Felix Beckmann, Julian Moosmann, Thomas O'Hallaran, Roger J. Guillory, D.C. Florian Wieland, Berit Zeller-Plumhoff, Regine Willumeit-Römer

**Affiliations:** a Institute of Metallic Biomaterials, Helmholtz Zentrum Hereon, Geesthacht, Germany; b Institute of Materials Physiscs, Helmholtz Zentrum Hereon, Geesthacht, Germany; c Deutsches Elektronen-Synchrotron DESY, Hamburg, Germany; d Department of Prosthodontics, Faculty of Odontology, University of Malmö, Malmö, Sweden; e Institute of Coastal Environmental Chemistry, Helmholtz Zentrum Hereon, Geesthacht, Germany; f Molecular Imaging North Competence Center, Kiel University, Kiel, Germany; g The Department of Oral and Maxillofacial Surgery Campus Kiel, UKSH, Kiel, Germany; h Department of Biomedical Engineering, Michigan Technological University, USA; i Department of Microbiology and Biochemistry, Michigan State University, USA; j Joint Department of Biomedical Engineering, Medical College of Wisconsin, USA; k Institute of Materials Mechanics, Helmholtz Zentrum Hereon, Geesthacht, Germany

**Keywords:** Biodegradable implants, Bone ultrastructure, Degradation, Mg-based alloys

## Abstract

Magnesium (Mg) – based alloys are becoming attractive materials for medical applications as temporary bone implants for support of fracture healing, e.g. as a suture anchor. Due to their mechanical properties and biocompatibility, they may replace titanium or stainless-steel implants, commonly used in orthopedic field. Nevertheless, patient safety has to be assured by finding a long-term balance between metal degradation, osseointegration, bone ultrastructure adaptation and element distribution in organs. In order to determine the implant behavior and its influence on bone and tissues, we investigated two Mg alloys with gadolinium contents of 5 and 10 wt percent in comparison to permanent materials titanium and polyether ether ketone. The implants were present in rat tibia for 10, 20 and 32 weeks before sacrifice of the animal. Synchrotron radiation-based micro computed tomography enables the distinction of features like residual metal, degradation layer and bone structure. Additionally, X-ray diffraction and X-ray fluorescence yield information on parameters describing the bone ultrastructure and elemental composition at the bone-to-implant interface. Finally, with element specific mass spectrometry, the elements and their accumulation in the main organs and tissues are traced. The results show that Mg-xGd implants degrade *in vivo* under the formation of a stable degradation layer with bone remodeling similar to that of Ti after 10 weeks. No accumulation of Mg and Gd was observed in selected organs, except for the interfacial bone after 8 months of healing. Thus, we confirm that Mg-5Gd and Mg-10Gd are suitable material choices for bone implants.

## Highlights

• Mg-based implants with Gd contents of 5 and 10 wt % degrade with the formation of a stable degradation layer.
• Mg-xGd and Ti implants result tight bone-implant contact.
• No traceable amount of Gd in the bone around the implant.
• No effect of the Mg-xGd degradation on the HAp 002 crystallite size and the platelet thickness around implants was observed.
• Mg is homogeneously distributed among the organs with a slightly lower concentration in liver and brain.

## Graphical abstract

**Figure:**
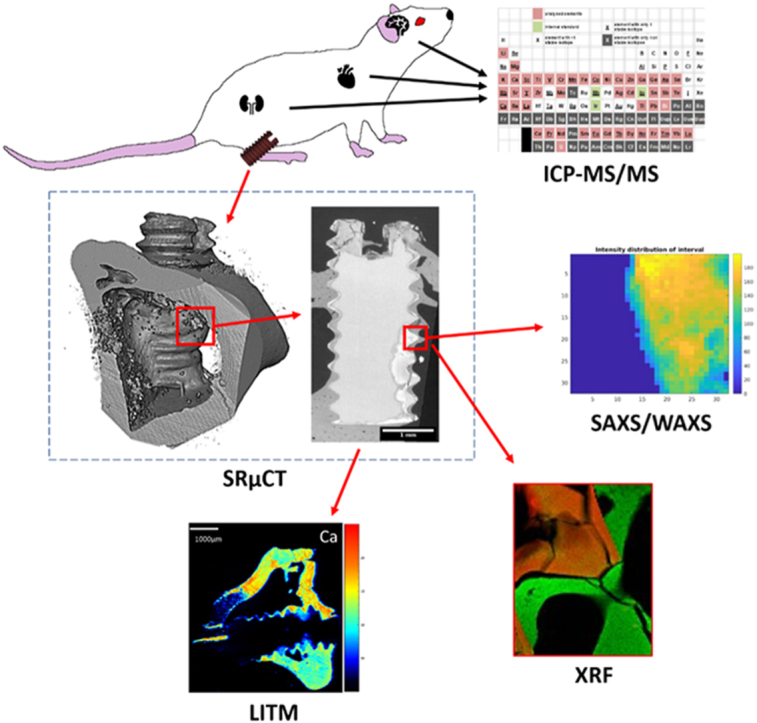

## 1 Introduction

Magnesium (Mg) and its alloys are a highly relevant class of temporary implant materials for orthopedic applications due to their biodegradability and biocompatibility [1,2]. The mechanical properties of Mg-based alloys match natural bone better than conventional implant materials (titanium (Ti) or stainless steel). For example, Mg has an elastic modulus of 41 GPa–45 GPa, which is closer to bone, having an elastic modulus of 3 GPa–20 GPa as compared to Titanium with an elastic modulus of 100 GPa–117 GPa [3]. Consequently, this close match can reduce the risk of stress shielding and the associated loss of bone density [3]. Moreover, a second surgical intervention to remove the implant after bone fracture fixation is no longer necessary which minimizes patient pain, surgery risk and medical costs [4]. Mg-based implants are generally recognized as biocompatible with beneficial effects for new bone formation, immune response and cartilage tissue regeneration [5,6]. The formed corrosion products were shown to be excreted from the body system with urine [7,8].

To use Mg-based materials as implants, it is vital to tailor their degradation rate and mechanical properties. High degradation rates can lead to an early reduced mechanical integrity of the implant [9]. Further, the rapid degradation process results in hydrogen bubble formation and high pH, which can lead to cell damage [[10], [11], [12]]. In order to increase the degradation resistance of the implant and tailor its degradation rate, different routes can be used as surface modifications or alloying of the magnesium matrix [[13], [14], [15]]. For the alloying, the utilization of Rare Earth Elements (REE) such as gadolinium (Gd) in particular is intended to improve the corrosion resistance of Mg alloys, ensuring a more controlled and predictable degradation rate. The presence of REEs can significantly enhance the strength and hardness of magnesium alloys, making them more durable and better suited to withstand physiological loads over the necessary period of bone healing [13,14].

**Fig. 1:**
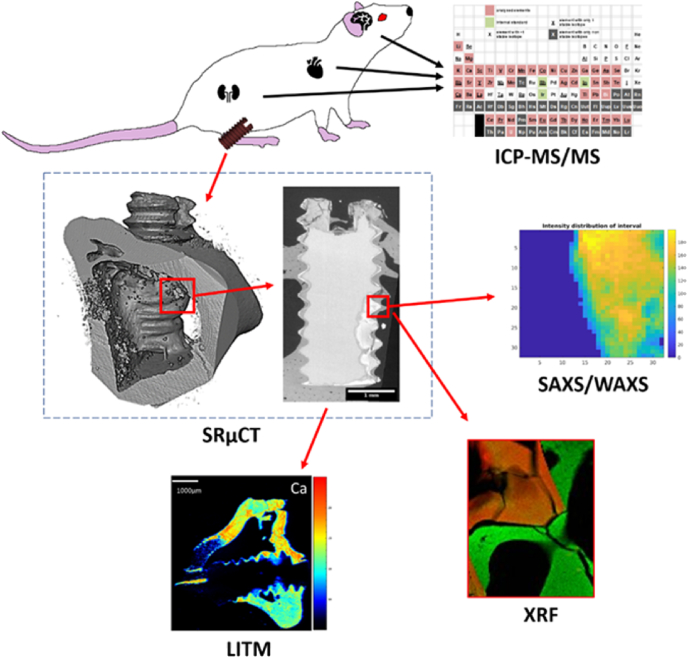
Schematic image showing the multi-modal nature of the study. Screws were implanted in the diaphysis of the rat's tibia. Top right: ICP-MS/MS - determination of element concentration in organs. From down left to right: SRμCT - volume rendering of a screw surrounded by bone and a cross section slice, SAXS/WAXS - 2D reconstructed intensity map of the (002) HAp reflex, XRF – elemental distribution map (green is bone, red is degradation layer) and LITM – high sensitivity element distribution analysis.

Previously, Krüger et al. investigated the degradation process of two Mg alloys with Mg-5wt.%Gd (Mg-5Gd) and Mg-10 wt%Gd (Mg-10Gd) *in vitro*, demonstrating lower degradation rates and better texture of the latter [16]. In a corresponding *ex vivo* study, Krüger et al. confirmed the faster degradation of Mg-5Gd in comparison to Mg-10Gd in a rat animal model [17]. They also showed that the average bone-to-implant contact of the Mg-xGd alloy implants was comparable to the Ti control. However, the study was limited to 12 weeks of healing missing the information of the mid-term degradation. In this study the biological response of these materials was investigated as well, showing that Mg-5Gd had the highest osteoclast activity visible 4 weeks after healing in comparison to the other materials (PEEK, Ti and Mg-10Gd) studied [17]. Furthermore, Galli et al. performed gene expression analysis showing that the marker BMP6 was up-regulated in the presence of Mg, which was also visible by an increased bone volume as compared to the Ti control [18]. Zeller-Plumhoff et al. performed the investigation of the bone ultrastructure for the same samples [19]. They outlined the smaller hydroxyapatite (HAp) lattice spacing and crystallite size for the (310) reflex around Mg-5Gd and Mg-10Gd implants in comparison to Ti. They suggested the Mg or Gd incorporation into the ultrastructure as a possible factor inducing the change in the ultrastructure. However, no elemental analysis was performed to inspect the ionic substitution hypothesis further. In addition to the substitution, several recent studies suggest differences in the ultrastructure of the bone may be due to the fact that the newly formed bone around the Mg-based implants experiences less remodeling in comparison to Ti [20,21]. Mid-term studies of the bone ultrastructure response are missing to date. Apart from the bone response, a main concern for Mg-REE alloys is their metabolizing and, connected to this, accumulation in internal organs and cytotoxicity [22]. The work of Myrissa et al. documented an enrichment of Gd especially in the spleen but also in other organs over a time period of 36 weeks [23]. Studies investigating the cytotoxicity of Mg–Gd alloys *in vitro* didn't find any indication of a negative impact [24,25]. Additionally, it was reported by Peruzzi et al. in an *ex vivo* study that Gd remains at the Mg–Gd implant site and does not lead to a measurable amount in internal organs after 12 weeks of healing [26]. Overall, the effect of Mg–Gd alloy implants on bone healing and remodeling and the distribution of Gd in the body at longer healing times remains unknown. Due to the slow degradation rates of Mg-5Gd and Mg-10Gd, we expect no toxic range of the released ions. *In vitro* studies with different cell types showed a survival of cells above concentrations of 2 mM Gd [27]. The objective of this *ex vivo* study is to investigate the mid-term effect of Mg-xGd alloy implants on the bone structure at micro and nanoscale levels and the elemental distribution at the implant side and organs, see Fig. 1. This study evaluates the performance of biodegradable Mg-5Gd and Mg-10Gd implants in comparison to permanent implants made from Ti and polyether ether ketone (PEEK) across the micrometer and nanometer scale of bone hierarchy. It focuses on the degradation behavior over a duration between 10 and 32 weeks of healing and the resulting osseointegration and ultrastructure adaptation, as well as the Gd distribution. The mm-μm scale information is accessed by the use of synchrotron radiation micro computed tomography (SRμCT) to determine degradation rate, bone-to-implant contact and bone volume to total volume. The findings were further expanded with the nanometer scale analysis covering the platelet thickness, lattice spacing and crystallite size of the HAp at the bone-to-implant interface. These features were studied with the use of small and wide-angle X-ray scattering (SAXS/WAXS). To better understand the implant life cycle the elemental distribution of Gd in the peri-implant bone and internal organ tissue was investigated using inductively coupled plasma tandem mass spectrometry (ICP-MS/MS), as well as Mg, Gd, Ca and P distribution in the peri-implant bone by use of laser ablation time-of-flight ICP-MS (LA-ICP-TOF-MS, LITM) and scanning X-ray fluorescence imaging (XRF). Additionally, the potential impact on the homeostasis of further essential elements was determined in different tissues.

## 2 Materials and methods

### 2.1 Material processing

In the current study two different Mg–Gd alloys, Mg-5Gd and Mg-10Gd, were studied, and Ti and PEEK were used as reference materials. The Mg-based alloys were fabricated by the Helmholtz-Zentrum Hereon (Geesthacht, Germany) while Ti and PEEK samples were provided by Promimic AB (Mölndal, Sweden). Mg-based alloys were produced by extrusion, turning and milling. The final implant shape was a screw with M2 thread, 4 mm in length, 2 mm in diameter and 0.5 × 0.5 mm slotted head. All the details about the material production and screw manufacturing procedures have been published in Krüger et al. [16].

### 2.2 Animal experiments

The animal experiments were conducted at the Molecular Imaging North Competence Centre (MOIN CC) Kiel under the approval number: V 241–26850/2017(74-6/17)) in the framework of the BMBF project MgBone (Projektnummer 05K16CGB). The implantation and anaesthetic protocols were similar to those described by Krüger et al. [17] and Zeller-Plumhoff et al. [19]. Screws were implanted in the diaphysis of the tibia of Sprague Dawley adult male rats. Two screws were implanted per animal, where one tibia received a Mg-5Gd or a Mg-10Gd screw, and the other tibia was used as control hosting either a screw made from Ti or PEEK. This experimental design allowed the separation of muscles in three different groups: I) close to the Mg-based screw, II) same leg but muscle without any direct contact to the implant, III) muscle at the control leg. In total 47 rats were used for this experiment because it was not always possible to analyze the pair of samples collected from the same animal due to the imaging artifacts and difficulties with sample preparation.

The animals were sacrificed 10, 20 and 32 weeks after implantation and cylindrical or box-shaped explants with a diameter/width of approx. 5 mm were cut from the tibia. As the animals were the subject of a multimodal study covering different aspects while minimizing the amount of animal needed, not all animals could be sacrificed at the exact same time points. The group denoted in the remainder of the manuscript as 10 weeks had healing times of 61 days and 67 days. The group of 20 weeks had healing times of 139 days and 148 days. The group denoted as 32 weeks was sacrificed after healing times of 202 days.

*Ex vivo* tissue and organ samples samples were taken immediately after euthanasia, snap frozen in liquid nitrogen and stored until analysis at −80 °C.

In order to allow for a better comparison of the results from former studies, an additional 4 week sample was used for the XRF study taken from a previous study [19]. Animal experiments from this previous study were conducted after ethical approval by the ethical committee at the Malmö/Lund regional board for animal research, Swedish Board of Agriculture, with the approval number DNR M 188-15.

The number of analyzed samples for each measurement, healing time point, and material is presented in Table 1. Details about the number of samples per tissue/organ can be found further in Table 4.

**Table 1:** Amount of samples investigated in each experiment.

Measurement	Time/weeks	Mg-5Gd	Mg-10Gd	Ti	PEEK
SRμCT	10	5	5	5	5
	20	5	5	5	5
	32	5	5	5	5
XRF	4	–	1	–	–
	10	–	1	1	–
	20	1	1	1	–
	32	–	1	–	–
SAXS/WAXS	10	3	3	3	–
	20	3	3	3	–
	32	2	2	2	–
LITM	10	–	1	1	–
	20	–	–	–	–
	32	–	1	1	–

### 2.3 Computed tomography data acquisition and analysis

#### 2.3.1 Micro computed tomography (μCT)

To ensure the machining quality and for later comparison with the *ex vivo* results, all Mg-xGd screws were imaged prior to implantation. Screws were scanned with the use of laboratory X-ray source (Phoenix—X-ray Nanotom; GE, Wunstorf, Germany). The operating voltage was set to 100 kV, the current to 70 μA and the pixel size after binning was approx. 2.6 μm. The data sets were segmented using thresholding in Avizo 2021.1 (FEI SAS, Thermo Scientific, France). PEEK and Ti screws were not measured prior to implantation because they were not expected to change their shape over time.

#### 2.3.2 Synchrotron radiation based micro computed tomography (SRμCT)

SRμCT imaging of explants was performed at different beamlines, specifically, at the P05 and P07 imaging beamlines at the PETRA III storage ring at the Deutsches Elektronen Synchrotron (DESY), Hamburg, Germany, and at the I13-2 Imaging Branchline at the Diamond Light Source (DLS), Didcot, United Kingdom. Samples after 10 and 20 weeks of healing were scanned at I13-2 with a pink beam profile, while samples after 32 weeks of healing were scanned at P05 and P07 with a monochromatic beam profile. The details regarding the scanning parameters, including, energies and pixel size are presented in Table 2.

**Table 2:** Experimental setting characteristics.

Beamline	Material	Energy / keV	Pixel size / *μ*m
**I13**–**2**	Mg-xGd, PEEK, Ti	peak 23, *σ* ≈ 3	1.2
**P05**	Mg-xGd, PEEK	35	0.92
**P05**	Mg-xGd, PEEK	51	1.28
**P07**	Ti	59	1.06

SRμCT was performed on frozen samples for 10 and 20-week healing times at I13-2 and embedded samples for the 32-week healing time at PETRA III. This was based on the scheduling of the beamtimes. The frozen samples were imaged under a continuous stream of nitrogen gas. Subsequently, the frozen samples and those explanted after 32 weeks were fixed and dehydrated in a graded series of ethanol (starting at 70 %). They were then embedded in Technovit 7200 methyl-methacrylate based resin (Hereaeus Kulzler, Germany) by LLS Rowiak GmbH (Hanover, Germany).

**Fig. 2:**
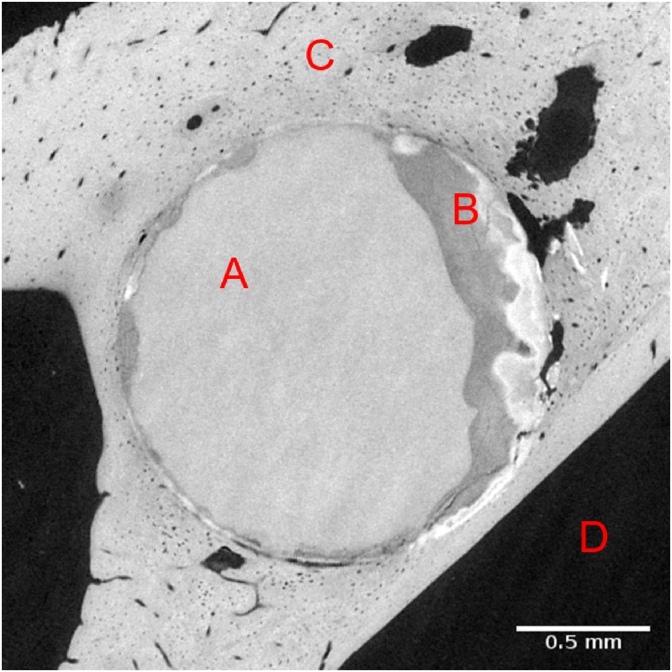
Tomographic cross section of Mg-5Gd screw in rat bone after 8 months of healing. Red letters show segmented features: (A) residual screw, (B) degradation layer, (C) bone and (D) background.

Data pre-processing and tomographic reconstruction were performed in a MATLAB-based framework (version 2020a, The MathWorks Inc., USA) [28,] with the ASTRA toolbox for tomographic backprojection of the data obtained at PETRA III and using the open-source Savu framework with the TomoPy reconstruction package [29,30] for DLS data. Prior to image segmentation the image volumes were resampled to 5 μm voxel size. By this it was guaranteed that all data had the same voxel size. The segmentation was performed with the use of a fully automated machine learning based segmentation framework developed by Baltruschat et al. [31]. Four material classes were distinguished: residual metal, degradation layer, bone and background (see Fig. 2).

The quantitative analysis was focused on parameters describing the degradation and osseointegration being degradation rate (DR, in mm/year) [32], bone-to-implant contact (BIC, in %) [] and bone volume to total volume (BV/TV, in %) [33], defined as

(1) $$DR=\frac{V_{i}-V_{r}}{A_{i}*t}$$

(2) $$BIC=\frac{A}{A_{d}}$$

(3) BVTV=VboneVbone+Vbackground

where V_*i*_ and V_*r*_ are the initial and residual volume of the screw, A_*i*_ is the initial surface area and t is the time of degradation. A is the contact surface area between degraded implant and bone, A_*d*_ is the surface of degraded implant, V_*bone*_ and V_*background*_ are the total number of bone voxels and background voxels in a region-of-interest (ROI) around the implant. The ROI was determined separately for each analyzed sample by enlarging a registered pre-implantation screw by 200 μm [17]. For better understanding the determination of BV/TV, please see Fig. 10 in the Supporting Information, where the bone ROI was visualized. Matlab R2022b (The MathWorks Inc., USA) was used to perform a two-way analysis of variance (ANOVA) for the statistical evaluation of the bone morphology. Only significant differences (p < 0.05) between either different materials at the same time point or the same material at different time points will be reported in the following.

### 2.4 Scanning XRF

For the scanning XRF and SAXS/WAXS experiments the explant blocks were first embedded into MMA using standard protocols by LLS Rowiak afterwards, the blocks were cut into halves along the screw manually. A 0.2 mm diamond band saw (Exakt Saw 300 CL, Exakt technologies, Inc., OK, USA) was used for this purpose. After that, one half per sample was laser cut by LLS Rowiak LaserLabSolutions GbmH (Hanover, Germany) into 10 μm thin sections. The sections were fixated onto Kapton tape. Thin sections were prepared for Mg-10Gd, Mg-5Gd and Ti samples only. A previous study of similar samples [19] showed no significant differences for PEEK samples in the bone ultrastructure to the other groups but showed Mg-xGd and Ti having different HAp crystal parameters. Therefore, PEEK samples were omitted in the ultrastructure study. While the given sample size for XRF measurements is low due to technical limitations, conclusions may be drawn in the context of the other data.

The scanning XRF study was performed at the i14 X-ray Nanoprobe beamline [34] at DLS. The measurements were performed in three beamtimes. An X-ray beam with an energy of 18 keV was used at the first beamtime and 10 keV at the later experiments, to improve the signal-to-noise ratio. The beam size was set to 100 × 100 nm^2^. For the XRF imaging, the Merlin Quad (QuantumDetectors, Oxford, UK) detector was used. Regions of 100 × 100 μm^2^ with 0.03 s of acquisition time per point were scanned. Three regions per sample were scanned: two at the interface of the cortical bone and degradation layer and one control of the cortical bone further from the interface. Due to limited beamtime, the XRF study focused on Mg-10Gd samples, as they have a higher percentage of rare earth elements in contrast to Mg-5Gd. For comparison, the Ti samples were studied. The used timepoints are 4, 10, 20 and 32 weeks. From the XRF measurement, two dimensional maps of the calcium (Ca), Gd, and phosphorus (P) distribution were constructed and analyzed. The Mg distribution in the bone could not be accessed as the wavelength of the Mg K_*α*_ line is at 1.2 keV was not detectable for the used setup as the measurements were performed at atmospheric conditions leading to total absorption by air of such low energies [35]. However, in order to have an indirect measure for possible change of the HAp composition we determined the calcium to phosphorus ratio.

For the XRF analysis, the fluorescence maps were first reconstructed using the DAWN 2.18.0 software (Diamond Light Source, Didcot, UK) [36,37] and then saved three elemental maps per ROI (Gd, Ca, P) into respective.tiff files. After that, using MATLAB R2018a (The MathWorks, Inc., Natick, US), the elemental maps were segmented into bone, degradation layer, and empty space on the basis of Ca (bone) and Gd (implant) presence. Based on the segmented maps, the Ca/P ratio and its dependence on the distance to the implant interface were analyzed. At the same time, elemental ratios within the degradation layer of the screw were analyzed. A statistical evaluation of the elemental ratios was performed in MATLAB using the *t*-test.

### 2.5 Scanning SAXS/WAXS

Scanning SAXS/WAXS measurements were performed at the P03 nanofocus endstation at the PETRA III at DESY [38,39]. The energy of the X-ray beam was set at 15 keV with the beamsize of 1.5 μm × 1.5 μm. To collect both SAXS and WAXS data, an Eiger 9 M (DECTRIS Ltd., Baden-Daettwil, Switzerland) detector was used. Thin sections of the explants containing Mg-5Gd, Mg-10Gd, and Ti implants after 10, 20 and 32 weeks of healing were studied. For each sample 90 × 90μm^2^ ROIs with the step size of 5 μm and acquisition time of 4s per point were scanned. Lanthanum hexaboride was used for the calibration of the SAXS/WAXS data. The ROIs at the bone/implant interface were located with the help of the build-in microscope. In order to normalize the scattering signal with respect to the beam intensity, a diode built into the beamline upstream was used.

Azimuthal data integration was done using PyFAI [40,41] python library (ESRF, Grenoble, France). Through PyFAI the dead and hot pixels were removed from the SAXS and WAXS images. The q-range was calibrated by applying the calibrant data obtained during the experiment. The HAp platelet thickness was calculated using the stack of cards model [42] by fitting the Kratky plot [43]. For the WAXS analysis, the position and full width at half maxima (FWHM) of the diffraction peak (002) was calculated by a Gaussian fit. In order to calculate the d-spacing for the (002) Bragg peak, Bragg's law

(4) *nλ* = 2*dsinθ*

where n is the diffraction order, *λ* is the X-ray wavelength, and *θ* is the glancing angle, was utilized. To estimate the crystallinity the Scherrer equation:

(5) $$\tau =\frac{K\lambda }{\beta \;\cos\;\theta }$$

where K is the shape factor close to 1, and *β* is the fitted Gaussian peak broadening, was applied [44]. A statistical evaluation of the bone ultrastructure between groups was performed in Matlab using the *t*-test.

### 2.6 Determination of element concentration in tissue samples

#### 2.6.1 Tissue sample preparation

For the elemental distribution analysis, we focused on the major excretion organs, the muscle tissue close to the implant and organs having indications for a Gd enrichment. Investigations were performed on the time points at 20 weeks and 32 weeks post-surgery. The frozen tissue samples were freeze dried (Gamma 1–16 LSCplus, Martin Christ Gefriertrocknungs-Anlagen GmbH, Osterode, Germany). The dried tissue was carefully minced and homogenized by a bead ruptor utilizing inert ceramic beads (Bead Ruptor 24 Elite, OMNI International, Kennesaw, USA). Brain tissue was not suitable for this procedure and, therefore, applied without milling for the element digestion. When available, triplicates (50 ± 5 mg dry weight) of each sample were weighted and transferred into 15 mL Digitubes(r). The digestion solution contained 0.5 mL H_2_O_2_, 1.0 mL HCl and 2.5 mL HNO_3_. The whole mixture was placed in a heating block for 2h at 80 °C (ANALAB, an Elemental Scientific Company, Hoenheim, F). Finally, the cooled digestion solutions were filled up to 15 mL with ultrapure water type 1. Reference materials made of mussel (BCR-668) and bovine liver (NBS-1577) tissue samples, as well as reagent blanks were treated accordingly in order to perform quality control of the analytical methods.

#### 2.6.2 Application of ICP-MS/MS

The elemental composition of the digested samples was analyzed by ICP-MS/MS utilizing an Agilent 8800 Triple Quadrupole MS (Agilent Technologies, Tokyo, Japan) coupled to an autosampler (ESI SC 4 DX FAST, Elemental Scientific, Omaha, USA). The measurement parameters are listed in Table 3. The applied multielement method is based on standard procedures which were described elsewhere [45,46]. The spray chamber, as well as the skimmer cone, were cleaned regularly with ultrapure water. Furthermore, the instrument and the stability of signals were controlled and optimized with a certified tune solution on a daily basis. The quantification was performed utilizing multielement calibration standards prepared daily and covering a concentration range of 0.1–100.0 μg/L and 10–10000 μg/L, respectively (ICP multielement standard VI, Merck, Darmstadt, Germany). Additionally, blank controls containing the digestion solution, as well as routinely prepared recovery standards, were measured as quality control measures. Different collision and reaction cell gases were utilized to minimize interferences on certain targeted elements and their isotopes respectively. Data were processed and evaluated by using the Agilent mass hunter software (version 4.4) as well as a custom-made Excel template for the evaluation of the multi elemental data. Outliers and values below the limit of detection were removed and the limit of quantification was calculated. Statistical evaluation of the tissue elemental distribution was performed utilizing the software Statistica (STATISTICA 13.1, StatSoft, Tulsa, USA).

**Table 3:** Technical conditions of ICP-MS/MS.

Parameters	Value and condition
Spray chamber	Double-Pass Scott Spray chamber
Temperature of spray chamber	2 °C
Nebulizer	ESI MicroFlow
Plasma-Vacuum-Interface	Cone made of Nickel (Ni); Skimmer cone made of Platinum (Pt)
RF-Power	1550 W
RF-Matching	1.80 V
Carrier gas volume flow	1.12 Lmin^−1^
Make-up gas volume flow	0.10–0.12 Lmin^−1^
Collision and reaction cell	No gas, H_2_, He, O_2_, H_2_HMI

**Table 4:** Mg and Gd distribution in selected organs determined using ICP-MS/MS. n.d. not determined, <LOD below limit of detection.

Tissue/organ	Mg / mg kg^−1^		Gd / μg kg^−1^	
N = (20 weeks/32 weeks)	20 weeks	32 weeks	20 weeks	32 weeks
Heart (17/16)	829 ± 68	900 ± 100	<LOD	1.0 ± 6.0
Liver (16/17)	716 ± 34	720 ± 60	1.0 ± 0.8	1.7 ± 0.7
Pancreas (8/17)	975 ± 135	824 ± 124	<LOD	<LOD
Spleen (8/16)	825 ± 124	590 ± 110	3.4 ± 2.4	6.0 ± 5.0
Kidney (16/16)	833 ± 107	810 ± 140	1.4 ± 1.1	3.0 ± 1.8
Brain (-/18)	n.d.	640 ± 90	n.d.	1.0 ± 6.0
Serum (-/17)	n.d.	29 ± 7	n.d.	<LOD
Muscle reference side (8/18)	960 ± 85	840 ± 240	<LOD	<LOD
Muscle PEEK + Ti (6/17)	810 ± 167	625 ± 170	3.1 ± 4.6	170 ± 445
Muscle Mg-5Gd (2/8)	703 ± 18	470 ± 240	600 ± 500	200 ± 300
Muscle Mg-10Gd (4/8)	610 ± 220	660 ± 170	130 ± 130	40 ± 70

### 2.7 High sensitivity element distribution analysis in bone tissue

#### 2.7.1 Application of LA-ICP-TOF-MS (LITM)

Laser-cut sections of bone tissues with a thickness of 10 μm were scanned with a TofWerk ICP-TOF-MS (LITM) custom system, which was coupled with a Bioimage 266 (Elemental Scientific Lasers) at a laser spot size between 10 μm and 20 μm. To achieve element standard curves/planes, gelatin standards prepared to known concentrations of Mg, Ca, Gd, and Ti salts were scanned with the tissue sections using the same instrument set-up. To prepare gelatin standards, mixtures that contain multiple elements were fully dispersed into 10 wt% gelatin to the final concentration ranging from approximately 10 PPM to 1000 PPM. The details of these steps are given in the supporting information. Section SI 1. Gelatin standards were cooled and cryosectioned to 10 μm thick to match bone tissue sections. Before and after scanning the tissue sections, the LITM system scanned all standards. In addition, gelatin standards were scanned three times in the middle of the tissue section scan with a pre-designated interval. The resulting timeline data that includes both the tissue section and the standard counts per second (CPS) signals were processed to interpolate the concentration of each quantified element.

#### 2.7.2 Data analysis

LITM raw data was processed and visualized by iolite 4 software in.io4 data format. The baseline was defined using a gelatin blank control scan in the “Total Beam” channel, before use in the data reduction scheme (DRS). For the PPM conversion, an element specific PPM was calculated based on specific standard scans using DRS in iolite 4. The channels (elements) of interest were selected for further data and imaging analysis. To determine locations of corroded implants, bone tissues, and other tissues, 26 Mg, 44Ca, 31P, 47Ti, 56Fe, and 155Gd maps were used, as implants contains contain high Mg and Gd or Ti, native bone tissue contains high Ca and P, and soft tissues contains high Fe. To select the degradation area, the following criteria should be met: 1. no tail streak due to inadequate washout of the ablation cell; 2. Gd or Ti signal is visible when upper pixel value limits were set to be the 99.9 % percentile; 3. Ca and P signals are less than 50 % of its 99.9 % cut-off signal; 4. Fe signal is lower than 20 % of its 99.9 % cut-off signal. The vicinity tissues were selected based on their distances to the implant-bone interface. A control bone tissue area was also selected as a reference. Examples of the areas of interest are shown in Supplementary Fig. 1. An area that excludes but is close to the bone tissue was selected as the instrument baseline control area, from which the limit of detection of each channel was determined by using:

(6) *LOD* = *mean* + 3 × *s*

where *LOD* is the limit of detection of the designated channel; *mean* refers to the average signal of the specific channel in the control area; *s* indicates sample standard deviation of the corresponding channel in the control area. To perform further analysis, CPS and PPM signals of Mg, Ca, Gd and Ti channels, along with CPS of ^31^P and ^44^C, were exported by regions. Data was visualized by using R (version 4.3.2) programming with the following packages: ggplot2, rgl and html widgets.

## 3 Results

### 3.1 Bone microstructure

#### 3.1.1 Degradation rate (DR)

**Fig. 3:**
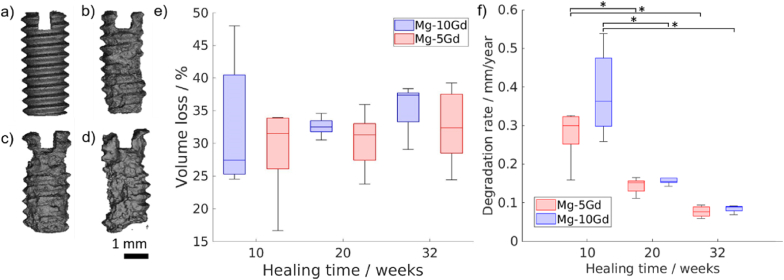
Visualization of residual material based on one representative Mg-5Gd sample for a) - pre-implantation screw, and residual screw after b) 10 weeks, c) 20 weeks and d) 32 weeks of degradation. e) volume loss and f) degradation rates values quantified for Mg-5Gd and Mg-10Gd after 10, 20 and 32 weeks of healing; sample size was 5 samples per material per timepoint, * indicates p < 0.05 using two-way ANOVA.

The *ex vivo* degradation rates for Mg-5Gd and Mg-10Gd implants dermined from SRμCT imaging are presented in Fig. 3. No significant differences were observed between both materials after degradation for the same time. Between 10 and 20 weeks, DR values are decreasing significantly (p < 0.05) for both alloys and after 32 weeks they reach a value of approximately 0.08 mm/year. Subfigures (a)–(d) of Fig. 3 show the volume renderings of the residual metal for Mg-5Gd samples to visualise the material loss over the healing time. The threads are losing their sharpness, and the metal is slowly diminishing, but the forming degradation layer (see Figs. 2 and Supplementary Fig. 12 in the Supplementary Information) is stable and the screw shape is still recognizable even after 8 months of healing. See Table 5 in Supplementary Information for numeric values and Fig. 10 for visual comparison of degradation of Mg-5Gd and Mg-10Gs screws over the healing timepoints.

#### 3.1.2 Bone-to-implant contact (BIC)

**Fig. 4:**
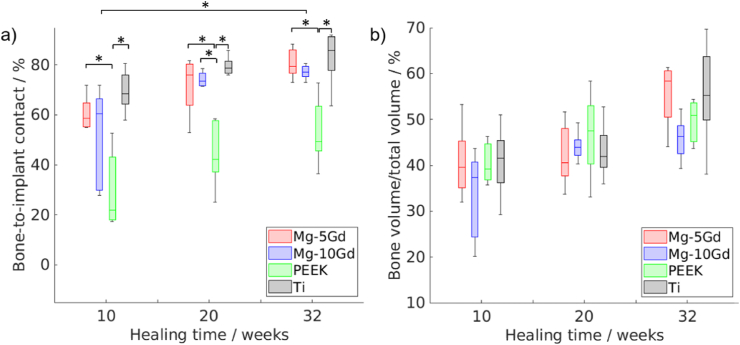
a) BIC and b) BV/TV values quantified for Mg-5Gd, Mg-10Gd, PEEK and Ti samples after 10, 20 and 32 weeks of healing. BV/TV was quantified in a distance of 200 μm away from the surface of pre-implantation screw. For numeric values see Table 5 in the supplemental material. Sample size: 5 samples per material per timepoint, * indicates p < 0.05 using two-way ANOVA.

The BIC parameter describes the percentage of the implant screw being covered by mineralized bone and, thus, is a direct measure for the osseointegration. The BIC values were determined from the SRμCT scans and show an increasing connection of all implant materials over time, see Fig. 4a. For better visualization of BIC, the representative images were added to Fig. 12 in the Supplementary section. The behaviour of both Mg-xGd alloys was similar at each time point, with BIC increasing from approx. 50-60 %–80 %. Differences in BIC for the same material over time were only significant (p < 0.05) for Mg-10Gd between 10 and 32 weeks. Slightly higher BIC values were quantified for Ti samples, but the differences between Ti and Mg-xGd were not significant at each time point. Overall, the contact between bone and implant for Ti was already at a high level and the parameter increased only by 10 %. The lowest contact was observed for PEEK samples, which were significantly lower than the other three materials at every time point (p < 0.05), except for Mg-10Gd at 10 and 32 weeks. For PEEK, the average BIC increased from only approx. 30 % after 10 weeks to approx. 53 % after 32 weeks. For qualitative comprehension of BIC, see Fig. 12 in the Supplementary Information which presents longitudinal cross sections of *ex vivo* SRμCT scans of all materials at 32 weeks post implantation. A stable degradation layer for both Mg-based alloys can be observed, which generally creates a continuous connection with mineralized bone. The Ti samples also indicate a constant connection, in contrast to PEEK samples where gaps between implant and the bone were observed.

#### 3.1.3 Bone volume to total volume (BV/TV)

The quantified BV/TV results in a selected volume of interest 200 μm away from the surface of the screw are shown in the right graph of Fig. 4b. Overall, there are no significant differences between materials and time points - the mineralized bone volume stays at approx. 40 % (only Mg-10Gd after 10 weeks indicates 33.3 ± 9.9 % BV/TV) at 10 and 20 weeks post implantation. After 32 weeks of healing the bone volume is slightly increasing for all investigated materials and is the highest for Mg5Gd and Ti (approx.55 %). Bone growth is observed especially between the threads and also inside the slotted screw head.

### 3.2 Bone ultrastructure

#### 3.2.1 Elemental distribution

**Fig. 5:**
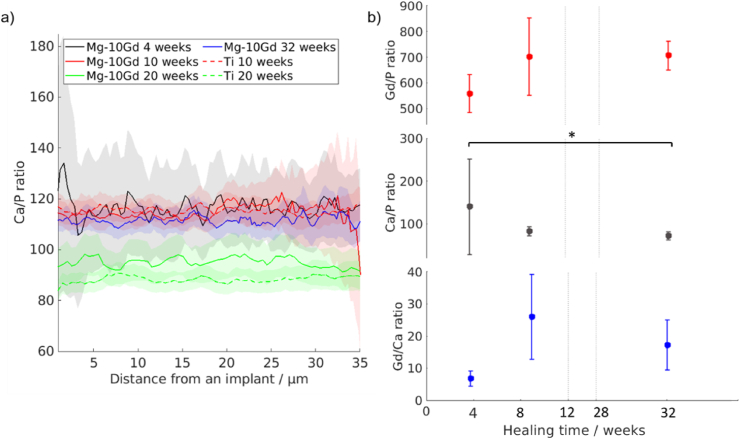
a) The Ca/P ratio as a function of the distance from the bone-to-implant interface. Solid lines represent Mg-10Gd alloy and the dotted line represents titanium. The lines represent the mean value, while the shade represents the confidence interval. b) Elemental ratios in the degradation layer of Mg-10Gd implant: Gd/P (red), Ca/P (black), and Gd/Ca (blue) after 4, 10, and 32 weeks of healing. Samples size: 7 samples, *t*-test, p < 0.05.

Fig. 5 depicts how the calculated Ca/P ratio depends on the distance from the bone-to-implant interface. Overall, the Ca/P ratio of both Mg-10Gd and Ti does not change in the area up to 30 μm from the implant. The decrease at close distance up to 2 μm to the implant was most likely caused by the segmentation imperfection of the XRF maps. The Ca/P ratio for Mg-10Gd after 4, 10, 20 and 32 weeks of healing did not show significant differences. Moreover, no Gd was traced outside the degradation layer. The 20 week time point for both Mg-10Gd, and Ti were measured during the first beamtime with the higher energy in comparison to other groups, which led to slightly lower values in terms of Ca/P ratio.

**Fig. 6:**
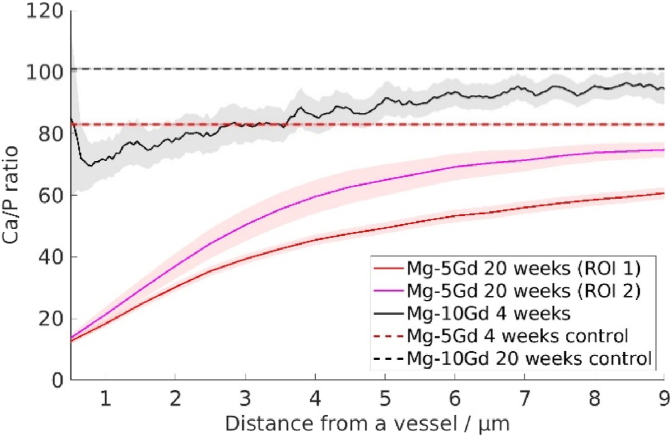
The Ca/P ratio in the bone tissue surrounding a blood vessel. The black solid line represents the mean Ca/P ratio for Mg-10Gd treatment after 4 weeks of healing, the red lines Mg-5Gd treatment after 20 weeks. The shade represents the confidence interval. Mean values calculated in the control regions are indicated with dashed lines. Samples size: 3 samples per materials for 10 weeks and 20 weeks, 2 samples per materials for 10 weeks and 20 weeks, *t*-test, p < 0.05.

Additionally, Ca/P, Ca/Gd, and Gd/P ratios were calculated within the degradation layer in order to see if the elemental composition of the layer changes with time. The 20 weeks timepoint was excluded due to the inability to account for the difference in fluorescence intensity caused by the different X-ray beam energy. Overall, the Ca/P ratios were lower for the degradation layer in comparison to the bone tissue around the implant, as shown in Fig. 5. The XRF analysis of the degradation layer demonstrated statistically significant difference (p < 0.05) only in Gd/P ratio between 4 and 32 weeks, revealing it to increase with time. The Gd distribution map demonstrates the bright Gd-containing particles in the degradation layer at every time point, as Fig. 13 shows. In addition to the ROIs at the bone/implant interface, ROIs surrounding blood vessels in the bone were investigated and compared to control regions. Two vessels were studied for a Mg-5Gd containing sample after 20 weeks of healing and one vessel of a Mg-10Gd containing sample after 4 weeks of healing, all of the vessels were around 50 μm away from the bone/implant interface. Fig. 6 demonstrates the Ca/P ratio as a function of a distance from the blood vessel wall.

#### 3.2.2 Platelet thickness

**Fig. 7:**
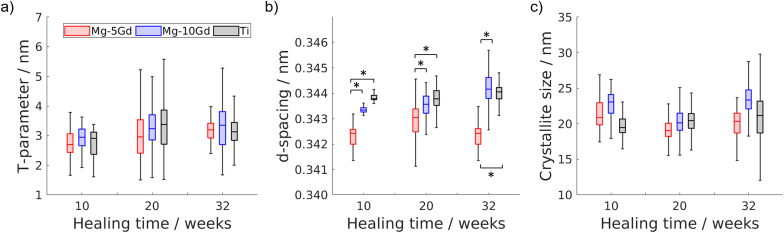
a) The average HAp platelet thickness, b) the average computed (002) d-spacing, and c) the average computed (002) crystallite size of the Mg-10Gd (blue), Mg5Gd (red), and Ti (black) after 10, 20, and 32 weeks of healing. The graph represents the median value, lower and upper quarterlies (notch), and maximum and minimum values (whiskers).

The boxplots demonstrating the median values of the platelet thickness for each group, as well as lower and upper quartiles, and maximum and minimum values, are shown in Fig. 7a. The median values of the T-parameter varied between 2.7 and 3.5 nm, which is similar to previously reported 3.2–3.6 nm for the same animal model [19], but slightly higher than the reported 1–2 nm by Liebi et al. [47]. The platelet thickness, as it is seen in Fig. 7a, did not show significant differences between the materials and time points. It is worth noting that the confidence intervals for the platelet thickness were high.

#### 3.2.3 D-spacing

The boxplots showing the median values of the (002) d-spacing for each group, as well as lower and upper quartiles, and maximum and minimum values, are shown in Fig. 7b. The lattice spacing of the (002) reflex demonstrated no significant difference between the Mg-10Gd and Ti groups at all the given times. The lattice spacing for Mg-5Gd (median value 0.342–0.343 nm), on the other hand, was significantly lower (p < 0.05) than both Ti (median value 0.343–0.344 nm) and Mg-10Gd (median value 0.343–0.345 nm) groups at all time points. There was no significant difference observed between the healing times for each material.

#### 3.2.4 Crystallite size

The boxplots demonstrating the median values of the (002) crystallite size for each group, as well as lower and upper quartiles, and maximum and minimum values, are shown in Fig. 7c. Similarly to the platelet thickness, the HAp (002) crystallite size did not show any significant changes between the materials nor healing time points. The median values of the (002) crystallite size varied between 15 and 30 nm. These values are very similar to the above-mentioned work of Grünewald et al., who reported the crystallite values of 10–18 nm around WZ21 implant in rat after 18 months of healing [48].

### 3.3 Inorganic element distribution within the tissue

The analyzed organs encompass excretion organs such as kidney and liver, as well as sensitive organs such as brain, heart, spleen and pancreas. Furthermore, muscle tissue close to the implant and from reference sides were investigated. There were no significant differences and correlations found in the concentration of the determined macro as well as trace elements such as potassium, calcium, iron, copper, manganese, zinc and selenium, regarding implant material as well as time points, as shown in Supplementary Tables 6, 7, 8, and 9 in the supporting information. Therefore, only the pattern of Mg and Gd are described in detail in the following sections and documented in Table 4 and graphically in Fig. 14 in the supporting information. In general, the results exhibit very high intra-individual variation among the animals and, therefore, a high standard deviation. Furthermore, the values for Gd have been measured in the range of the limit of detection which also contributes to a higher uncertainty. Consequently, the results were not feasible for a statistical evaluation and must be regarded as trends.

#### 3.3.1 Pattern of magnesium distribution

Mg is homogeneously distributed among the organs with a slightly lower concentration in liver and brain. There are no indications for a change in concentration comparing the two sampling time points, except for the spleen showing a slight reduction from 20 to 32 weeks.

The concentration in the muscle in close vicinity to the implant is of special interest. Therefore, three different types of muscle samples have been compared as described in the methods section. In general, a lower Mg concentration was observed in muscles close to Mg-xGd implants compared to muscles from the reference side at the other leg. A significant time dependency was not seen. The higher Mg concentration in muscle of the reference side might be due to an undisturbed Mg homeostasis but needs verification by analyzing untreated animals in order to set a baseline for tissue Mg distribution.

#### 3.3.2 Pattern of gadolinium distribution

A reliable determination of the Gd concentration was not possible for most of the tissues as the Gd concentration was at the edge of the instruments sensitivity along with complex composition of organic matrix increasing the difficulty further. There were indications for an increased concentration in the kidney and spleen over time, but this was due to high intra-individual differences, where only one animal has exceptionally high contents. In contrast to the Mg distribution, there is a remarkably higher concentration in the muscles close to the Mg-xGd implant especially after 20 weeks of implantation, with a slight decrease towards 32 weeks.

### 3.4 High sensitivity element distribution analysis in bone tissue

**Fig. 8:**
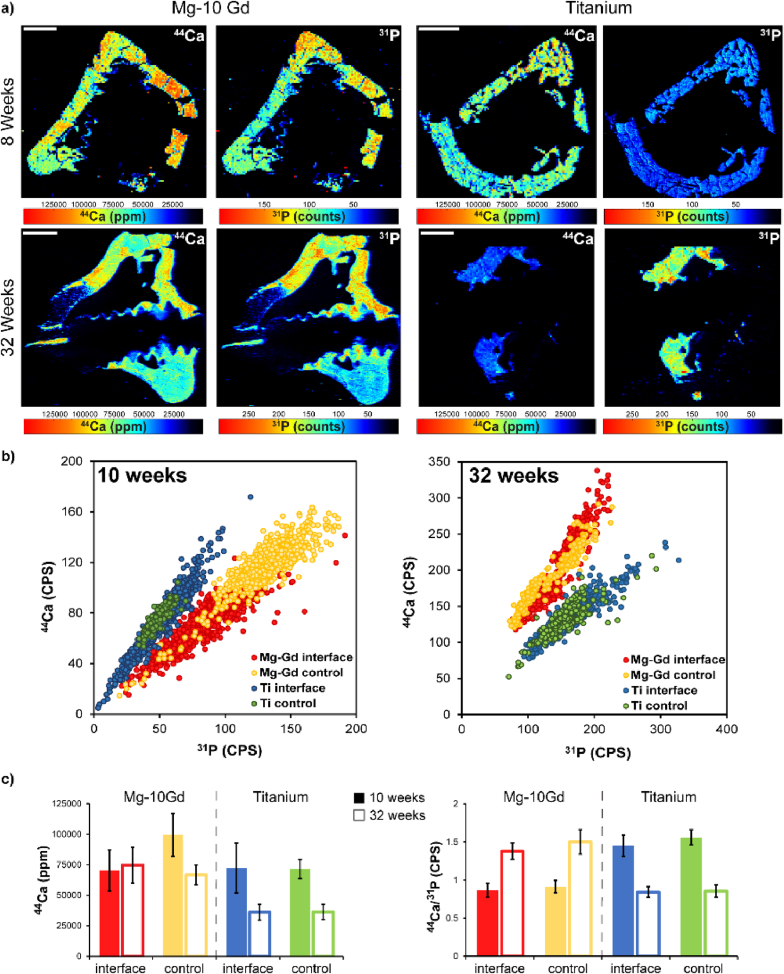
LITM scans demonstrating Ca and P presence of Mg-10Gd implants and titanium implants at 61 days and 8 months. a) shows Mg-10Gd and Ti implants at 10 weeks and 32 weeks respectively. b) demonstrates Ca vs. P on a per-pixel basis at 10 weeks and 32 weeks, respectively, of regions outlined from each material. c) distribution of elements and Ca/P ratios for the investigated areas.

**Fig. 9:**
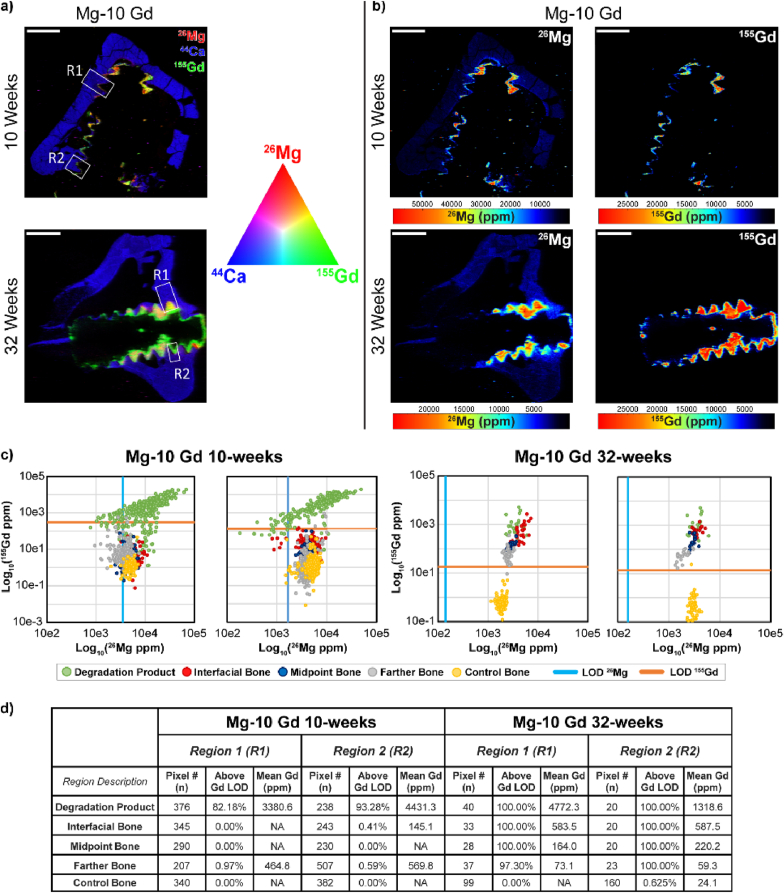
LITM scans demonstrating Mg and Gd presence of Mg-10Gd implants at 10 weeks and 32 weeks. a and b) show Mg-10Gd implants at 10 weeks and 32 weeks, respectively. Panel a represents triple overlaid scans containing Gd (green), Mg (red), and Ca (blue) mapping. c) shows region plotting of Gd vs. Mg, with corresponding to R1 and R2 in panel a) top and corresponding to R1 and R2 in panel a) bottom. The LOD for both Mg and Gd are plotted as vertical and horizontal lines.

LITM imaging was performed for the Mg-10Gd and Ti implants at 10 weeks and 32 weeks. Fig. 8 demonstrates these results in the context of ^44^Ca and ^31^P scans (Fig. 8 a-c). It should be noted that while Ca was able to be quantitated against a gelatin standard, ^31^P was not able to be quantified. Therefore, Ca signals for the images shown in Fig. 8 are scaled in ppm, and the ^31^P images are given in cps. Plotting of the ratio of ^44^Ca and ^31^P was done in cps for Fig. 8 b. While the approximately linear relationship of ^44^Ca and ^31^P was kept for Mg-10Gd screws vs. Ti screws at 10 weeks, it appears that ^31^P signal at the interface of Mg-10Gd screws was higher than at the interface of Ti screws. Interestingly, this relationship switches at 32 weeks (Fig. 8 panel b, right), with pixels at the interface of the Mg-10Gd screws showing a higher overall ^44^Ca signal when compared to Ti implants. We were interested in the Mg and Gd distribution near the implant at 61 days and 8 months and detailed our findings in Fig. 9. A detailed demonstration of region selection can be found in Supplementary Fig. 9, where pixels were identified in respective regions shown in Fig. 9 a, b and plotted in Fig. 9 c. It should be noted that the resulting images are linearly scaled, and approximately describe the upper 95 % of all pixel values for the respective element. Dark pixels do not necessarily equal a lack of signal, and this concept is demonstrated in Supplementary Fig. 17, where logarithmically scaled images are shown for the Mg-10Gd implants. The limits of detection were plotted for Mg and Gd on the plots given in Fig. 9 c. Control bone tissue was largely seen to have detectable Mg and non-detectable Gd for both implants evaluated. At 10 weeks, the only detectable Gd in the regions selected were within the corrosion product. This changes at 32 weeks, where interfacial bone, and bone tissue further from the implant site was positive for detectable Gd. The % of pixels in each region that were above the LOD for Gd are shown in Fig. 9 d. Interestingly, 100 % of all pixels from each region outlined besides the control tissue possessed detectable Gd.

## 4 Discussion

The determined degradation and bone tissue parameters agree well with those obtained by Krüger et al. [17] and Bruns et al. [49] for the same materials, yet different time points. Supplementary Fig. 11 and table 5 in the appendix show the results from the current study and other's [17] in one graph, highlighting their overall agreement. The results presented in the current study for 10 weeks of healing are very close to those presented by Krüger et al. [17] for 12 weeks of healing. The relationship for Mg-5Gd and Mg-10Gd is inverted, however, yet the differences between the groups are statistically insignificant. The alloying of the Mg matrix shifts the electrochemical potential and, thus, reduces the degradation rate. Similar to Krüger et al. [17], we can, therefore, argue that the alloying of the Mg matrix with Gd leads to slow degradation rates under physiological conditions and, thus, low hydrogen releases. Furthermore, degradation products are directly deposited in the vicinity of the implant resembling the original shape of the screw. Most likely, these degradation products consist of insoluble Gd compounds. This agrees with other studies, where rare-earth containing Mg alloys had been studied over the course of 9 months in rabbit femur [50].

A promising approach to describe the degradation behavior of Mg-xGd implants was investigated by Baraghtheh et al. [51] where the authors developed a computational model as an alternative way of describing the gap between *in vitro* and *in vivo* material degradation. The model is based on the previous research by Krüger et al. [16,17] and simulations cover the degradation for up to 100 days (approx. 14 weeks, which covers the first time point of the data presented herein). The results on the degradation after 10 weeks of implantation presented in the present work correspond well with the simulation of Mg-xGd *in vivo* degradation, in each case the DR is approx. 0.4 mm/year. The time points (20 and 32 weeks of implantation) may be used for developing further and more precise models.

The gap between *in vitro* and *in vivo* degradation is also due to the complex environmental condition within a living organisms. It has been shown that inflammatory conditions might impact the corrosion of a CoCrMo Biometal Munoz et al. [52]. Furthermore, the adsorption of plasma and interstitial body fluid proteins that might be enriched under inflammatory conditions such as antibodies and inflammatory cytokines can influence the corrosion by forming a dense protection layer. Since the implantation side strongly impacts the *in vivo* corrosion where soft tissues reduce the degradation rate and intramedullary implantation leads to faster degradation the standardized transcortical placement of the screws was performed Reifenrath et al. [53]. Considering systemic effects, the kidney and gastrointestinal diseases may affect the Mg homeostasis in the body. Since 95 % of the daily filtered Mg is usually reabsorbed, renal failure can lead to Mg loss and hypomagnesaemia and consequently by a faster utilization of the Mg storage in bone. Since there was no difference in the level of physiological relevant elements we suppose no impact of the environment on the implant degradation.

Further analysis of combined short- and mid-term experiments highlights the osseointegration which is described by BIC and BV/TV parameters and is shown in Supplementary Fig. 11. The comparison of BIC values for 4 and 32 weeks of healing show that the contact for Mg-5Gd was tripled (from 25.0 % to 80.8 %) and doubled for Mg-10Gd (from 36.0 % to 77.1 %). It is well visible on the tomograms (see Supplementary Fig. 12), that bone is forming tightly around the implant, inside the threads and, for later time points also inside the screw's head. We note that the degradation homogeneity can hardly be judged from these images as these are only two-dimensional cuts not showing the full three-dimensional characteristics. The herein present results and the data from Krüger et al. [[16], [17]] show that Mg-xGd and Ti have comparable bone-implant contact, while the parameter observed for PEEK is significantly lower, for all investigated time points. A relatively high BIC for biodegradable materials was also observed in the work of Sefa et al. [50], where the authors compared Mg-10Gd, Mg–4Y-3Re and Mg–2Ag screws implanted in rabbit femur for 26 and 39 weeks. Sefa et al. observed BIC values between 45 and 56 % for all materials, regardless of the implantation time. They showed that the degraded screws were well anchored in the bone, which displayed matured morphology. However, the screw geometry differed from this study as did the implantation site as three degradable screws were implanted in one leg, and also the animal species. Furthermore, even the high resolution SRμCT analysis of the BIC parameter can be impaired by imaging limitations and artifacts [54] but also underestimated by the bone shrinkage during the critical point drying sample preparation [55].

The overview graph showing the presented data within this work and the data from Krüger et al. on the BV/TV results showed that the mineralized bone volume around Mg-based alloys increased twice from approx. 24 % to approx. 50 % between 4 and 32 weeks of healing (see Supplementary Fig. 11 and Supplementary Table 5) which means Mg-xGd supports new bone formation. Bone formed around PEEK samples indicate similar progress, while bone volume around Ti samples is slightly higher (from 48.8 % to 55.7 %). Quantifications based on new bone formation in the vicinity of implants are common while assessing the osseointegration, and were also performed by Sommer et al. [56]. The authors investigated *in vivo* and *ex vivo* degradation of ZX00 pins (Mg–Ca–Zn alloy) implanted in osteoporotic, old and juvenile rats for up to 24 weeks. SRμCT analysis was evaluated for the longest healing time, showing that BV/TV in an area up to 1 mm away from the implant is approx. 40 % for a juvenile healthy rat, which is similar to the results shown in this publication.

The XRF study of the bone tissue around the Mg-10Gd and Ti implants appears to demonstrate that there is no traceable amount of Gd in the bone around the implant, which indicates that there is no tractable amount of Gd being incorporated into the bone tissue, as it was previously hypothesized [19]. We have also shown that there is no significant change in the Ca/P ratio depending on the distance, which means that we did not detect the incorporation of Mg^2+^ ions in the bone at the bone-to-implant interface. It is known that Mg^2+^ may replace Ca^2+^ ions in the HAp lattice [19,48]. That means that if any incorporation would take place, it would decrease the Ca/P ratio in the areas with the bigger concentration of magnesium. On the other hand, the analysis of the bone around the blood vessels demonstrated the decrease of the Ca/P ratio depending on the distance to the blood vessel in both Mg-5Gd and Mg10Gd samples, as shown on Fig. 6. This might indicate the deposition of Mg in the areas around the vessels being incorporated into the HAp. The same behaviour was previously shown by Grünewald et al., who reported a higher level of Mg around blood vessels [57].

In addition to XRF we further investigated the distribution of Mg, Ca and Gd in the bone tissue by high-sensitive LITM on Ti and Mg-10Gd samples. This data shows some difference to the XRF, especially at 32 weeks, which is partially related to the fact that a much larger area of the sample with a much more sensitive technique was performed. In terms of Gd distribution, this analysis shows that at 10 weeks of healing, no Gd could be detected in the tissue directly proving that Gd is just located in the degradation area. In the inspected areas only the degradation layer shows pixels above the limit of detection, see Fig. 9. This changes for 32 weeks of healing as Gd accumulation can also be seen here in the area close to the bone with 100 % of the pixel being above the *LOD* but also in the control area 25 % of the pixels are being above the *LOD*. In terms of Gd concentration, we can estimate a decreasing concentration from 1318 ppm in the degradation layer to 60 ppm to the bone area with the largest distance. The concentration in the control area was 24.1 ppm. Interestingly, the Gd concentration in the degradation layer decreases from 4431.3 ppm at 10 weeks to 1316.3 ppm at 32 weeks. LITM further shows that increased Mg concentrations are present, but constricted to the interface implant region, see Fig. 9 and the level of Mg is not increased in the major bone area. The Ca/P ratio for the Mg-10Gd and Ti sample at the observation points does not change from the interface region to the control area which is in line with the XRF data showing no variation as function of the implant interface.

In addition to the morphological and compositional analysis, understanding the bone nanoadaptation process is one of the keys to improving stability and durability of an implant [58,59]. In our study, we demonstrated that there is no significant difference in the lattice spacing between the HAp around Mg-10Gd and Ti. That means that the amount of Mg^2+^ ions released from Mg-10Gd and registered by LA-ICP-TOF-MS was too low to significantly influence the HAp crytal structure or to be detected by the XRF measurements. The lattice spacing of the HAp around the Mg-5Gd implant, on the other hand, was significantly lower than Ti and Mg-10Gd at any given time point. This could be due to the slightly (yet not statistically significant) higher mean degradation rate of Mg-5Gd in comparison to Mg-10Gd, and consequently a higher degree of Mg incorporation. This indicates that the higher degradation rate causes stronger effect on the bone ultrastructure. To prove this further LA-ICP-TOF-MS investigation of the bone around Mg-5Gd implant would be required. Additionally, the scanned ROIs were slightly influenced by radiation damage, which was shown by the change in the bone tissue colour. An effect of this damage on the demonstrated results is not anticipated, however, due to all the ROIs receiving equal radiation dose. The crystallite size and platelet thickness as opposed to lattice spacing did not significantly differ for Mg-5Gd, Mg-10Gd and Ti. This does not coincide with other studies that hypothesized Mg incorporation leading to smaller platelet thickness, crystallite size, and d-spacing altogether [19,47,48,57]. It was clearly demonstrated by several studies that Mg-based biodegradable implants are able to alter the bone structure around it. Liebi et al. revealed the decrease in the HAp platelet thickness size around the ZX10 implants in rats using SAXS tensor tomography [47]. It was also previously shown by Grünewald et al., that the bone ultrastructure is affected by ZX50 and WZ21 implants degradation at the distance up to 20 μm in Sprague-Dawley rats [48,57]. They reported the released Mg^2+^ ions of the rapidly degrading ZX50 being accumulated at the cortical bone at the implant interface temporarily. For both implant types, Mg was otherwise stored in the bone surrounding blood vessels, bone cells and the bone marrow. They also reported high Mg levels in bone leading to the smaller HAp platelets, crystalline order, and lattice spacing (002 reflex). The reason behind our results not showing that behavior is most likely the big confidence interval with is up to 50 % and 100 % for crystallite size and platelet thickness respectively, which can be caused by animal variability and a relatively low amount of studied samples. Moreover, the degradation rates of ZX50 reported by Grünewald et al. were significantly higher than those in the current study. The observed decrease d-spacing in this study might also indicate less remodeled bone compared to other materials, as it was seen in a study of ZX00 implants in a sheep model [20]. Sefa et al. demonstrated the higher remodeling rates of the bone around Ti implants compared to Mg-10Gd implants between 4 and 12 weeks of healing in rats [21]. This was thought to relate to microcracks forming around Ti implants due to larger differences in mechanical properties between the implant and the surrounding bone, which accelerated bone remodeling. These results were corroborated by the difference in ultrastructure observed by Zeller-Plumhoff et al. [19] and mechanical properties of the bone studied by Bruns et al. [49]. Push-out test performed on screw implants showed that implant stability benefits from different bone morphological properties depending on the biomaterial. Titanium implants promoted rapid callus formation and exhibited a consistent monomodal strain profile. By contrast, the bone volume fraction around the Mg–Gd alloy implants was minimal near the implant interface, displaying less ordered strain transfer. As seen in these studies, as well as Krüger et al. [17], the differences in bone micro- and ultrastructure and its functional properties between Mg-xGd implants and Ti diminishes over time. Therefore, the lower differences between Mg-xGd and Ti observed in the present study may be a result of the assimilation of the remodeling state of the bone over time. In the future, further studies should explore the correlation between bone remodeling rates and its micro- and nanoadaption.

In order to clarify the question of Gd and other implant element enrichment in susceptible organs a multielement analysis was performed. In contrast to the findings of Myrissa et al. [23], an accumulation of either major implant component, i.e. Mg and Gd has not been documented for the selected organs. This might be due to the reduced degradation rate of the material and due to the smaller size of the implant. However, this study is in agreement with the analysis by Zhao et al. who did not detect Mg accumulation in different organs as well as Cho et al. investigating tissue around a Mg–Ca–Zn implant in rabbits [60,61]. Considering the high intra-individual variance, it is highly recommended to increase the number of biological replicates in the future, as well as to avoid any contamination by separating and cleaning the preparation tools. Furthermore, determining a baseline for major trace and implant elements for untreated animals should be established in advance. Considering the trends for an increased concentration of Gd in the muscle tissue close to the implant as well as in the brain, liver and spleen, an accumulation cannot be completely excluded. Nevertheless, we believe that a toxic effect of Gd is unlikely for the implants under investigation. Previous *in vitro* studies with different cell types showed a survival of cells above concentrations of 2 mM Gd [27,62,63]. In addition, direct contact of Mg-10Gd and osteoblast-like cells was shown to lead to an increased gene expression of osteogenic markers [64]. Moreover, the toxicity of Gd under discussion arises from Gd-containing contrast agents, which have a completely different chemical complexation [65]. Further sensitive analytical methods such as Laser Ablation ICP-MS for a high resolution mapping of organs or the application isotopically enriched magnesium implants should be used in the future. Thus, it would be possible to improve the outcome of complex *in vivo* studies and clearly elucidate the fate of metallic implant components [66,67].

There are some indications of an enrichment of Mg in the implant surrounding bone. Especially an initial, short term increase was observed [68]. However, this accumulation seems to be reduced after further implant degradation and there is no long-term effect on Mg distribution, as also documented with the present study. An enrichment in the muscle tissue around the implant can also be excluded. Correlating the micro and nanoscale analysis of bone regeneration shows that remodeling around Mg-xGd implants is driven by the formation of degradation layer and element distribution within it. Over the healing time, the degradation rate is decreasing and the stable degradation layer is formed.

## 5 Conclusions

In the presented study the mid-term degradation and osseointegration of Mg-5Gd, Mg-10Gd, PEEK, and Ti implants were analyzed with high-resolution imaging techniques. Quantified degradation rates for both Mg-based alloys were comparable to previous, short-term studies. Osseointegration and bone regeneration were compared with control materials and with available literature and the results show stable contact and progressive bone mineralization of the Mg-xGd implants. Especially the bone-implant contact was improved or comparable in comparison to conventional materials such as Ti and PEEK. The Mg^2+^ ions being released from the degrading Mg-xGd are being stored both in the bone at the bone/implant interface and in the blood vessel wall. No effect of the Mg-5Gd and Mg-10Gd degradation on the HAp 002 crystallite size and the platelet thickness around the implants was observed. We could also observe that Gd can be detected in low amounts in the interfacial bone after 32 weeks. Since there was no serious body burden of alloying elements this study underlines the biocompatibility of Mg-based materials, although a routine monitoring of elements in sensitive organs and tissue in close vicinity to the implant should be included in *in vivo* studies, perspectively.

## Ethics approval and consent to participate

The animal experiments were conducted at the Molecular Imaging North Competence Centre (MOIN CC) Kiel under the approval number: V 241–26850/2017(74-6/17) in the framework of the BMBF project MgBone (Projektnummer 05K16CGB).

## CRediT authorship contribution statement

**Kamila Iskhakova:** Writing – review & editing, Writing – original draft, Software, Investigation, Formal analysis. **Hanna Cwieka:** Writing – review & editing, Writing – original draft, Software, Investigation, Formal analysis. **Svenja Meers:** Investigation, Formal analysis. **Heike Helmholz:** Writing – review & editing, Writing – original draft, Formal analysis. **Anton Davydok:** Investigation. **Malte Storm:** Software, Investigation. **Ivo Matteo Baltruschat:** Software. **Silvia Galli:** Investigation, Conceptualization. **Daniel Pröfrock:** Writing – review & editing, Supervision, Investigation, Formal analysis. **Olga Will:** Investigation. **Mirko Gerle:** Investigation. **Timo Damm:** Investigation. **Sandra Sefa:** Investigation. **Weilue He:** Investigation. **Keith MacRenaris:** Investigation. **Malte Soujon:** Investigation. **Felix Beckmann:** Software. **Julian Moosmann:** Software, Investigation. **Thomas O'Hallaran:** Investigation. **Roger J. Guillory:** Investigation. **D.C. Florian Wieland:** Writing – review & editing, Supervision, Investigation, Conceptualization. **Berit Zeller-Plumhoff:** Writing – review & editing, Supervision, Investigation, Conceptualization. **Regine Willumeit-Römer:** Writing – review & editing, Supervision, Conceptualization.

## Declaration of competing interest

The authors declare that they have no known competing financial interests or personal relationships that could have appeared to influence the work reported in this paper.
