# Supplementary material for: Multi-modal investigation of the bone micro- and ultrastructure, and elemental distribution in the presence of Mg-xGd screws at mid-term healing stages
Source: Bioact Mater. 2024 Sep 3;41:657–71. doi: 10.1016/j.bioactmat.2024.07.019 (PMC11408010; doi:10.1016/j.bioactmat.2024.07.019)
Supplement: Multimedia component 1 [file mmc1.docx]

Supporting information


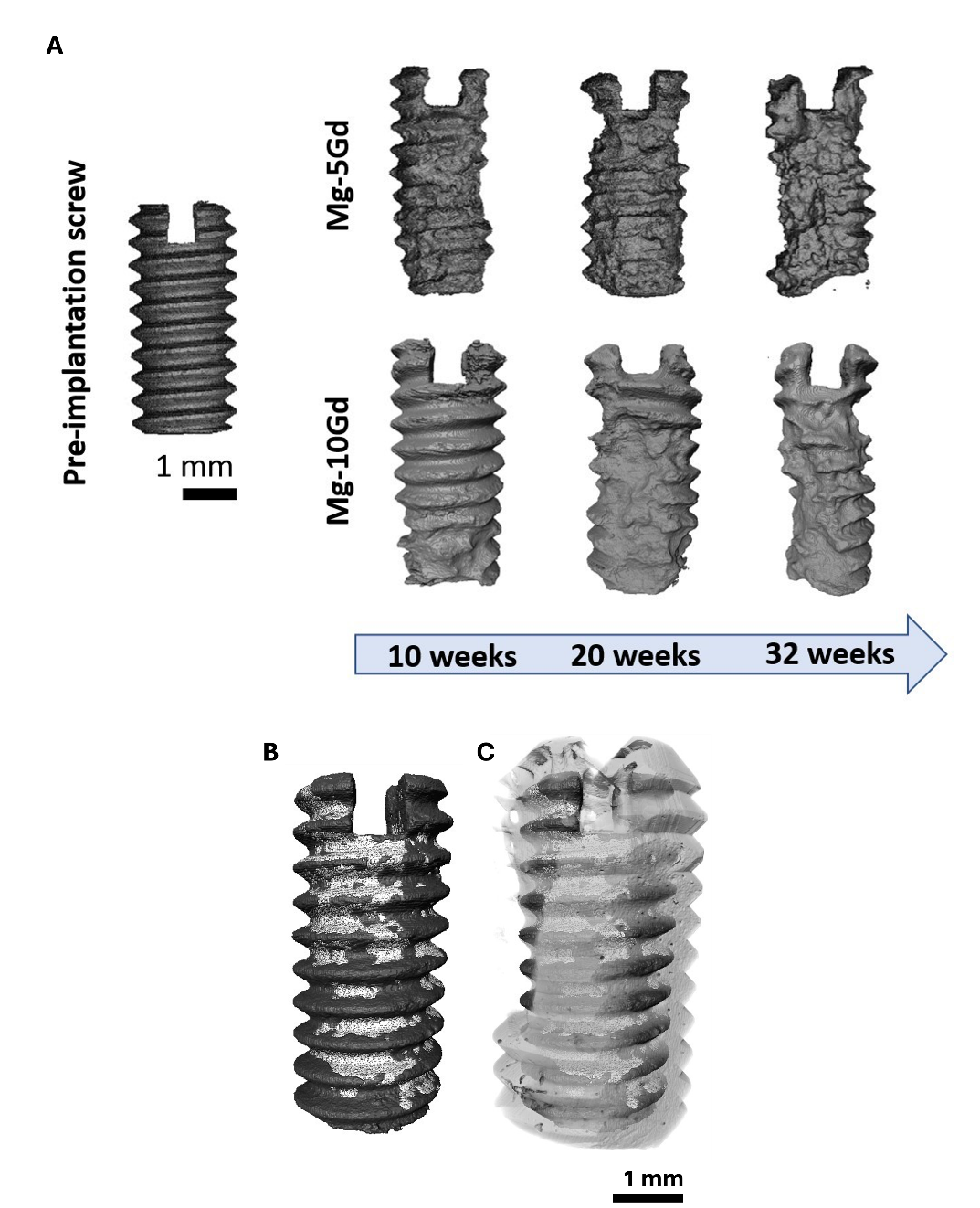


Figure 10: (A) Volume renderings of pre-implantation screw in comparison to residual metal of Mg-5Gd and Mg-10Gd screws to demonstrate the degradation of material in 10, 20 and 32 weeks. Mg-5Gd screw after 32 weeks of implantation: B) residual metal with degradation layer, C) bone ROI used for BV/TV quantifications.


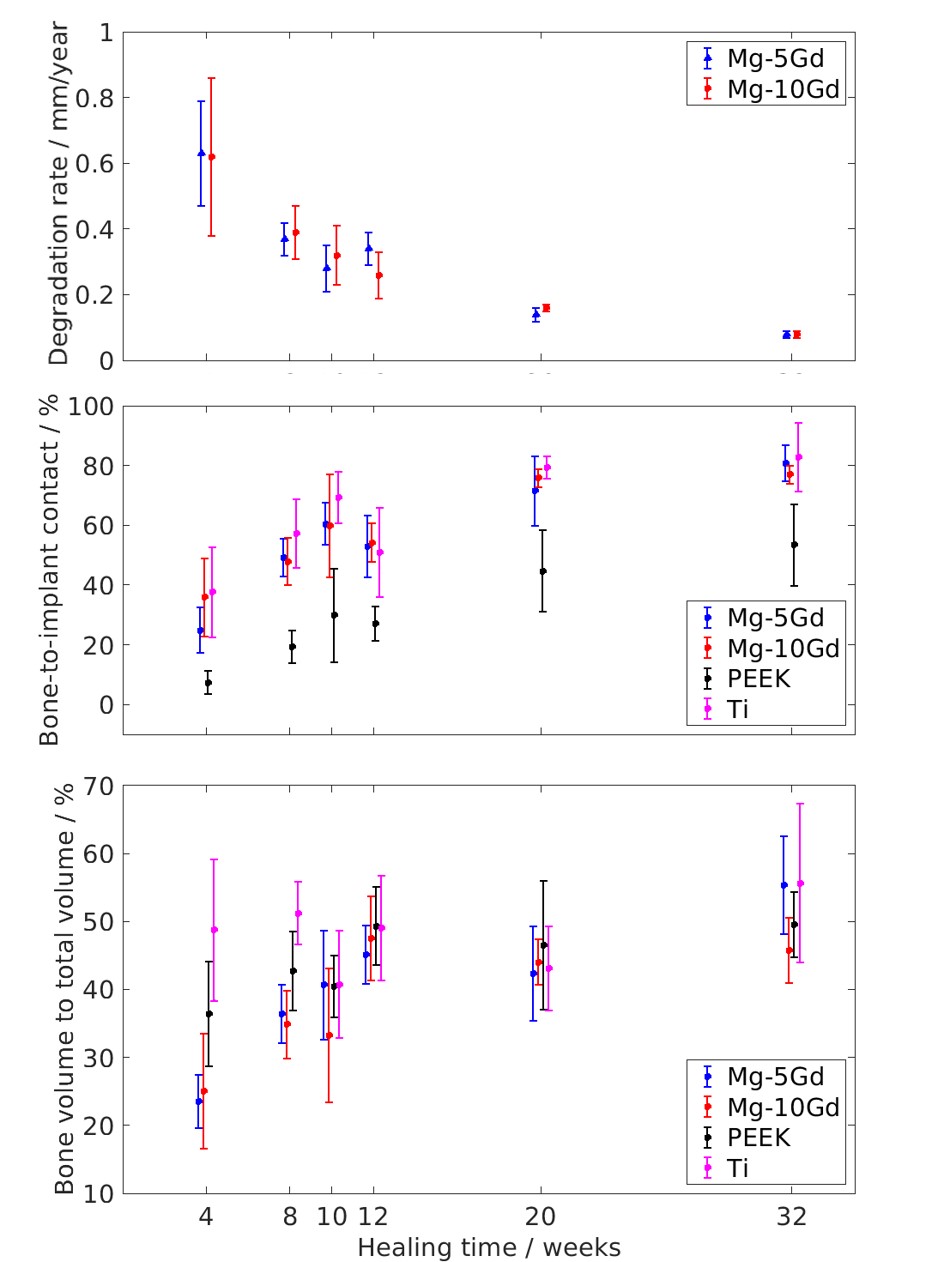


Figure 11: Comparison of DR, BIC and BV/TV values of Mg-5Gd, Mg-10Gd, PEEK and Ti explants after 4, 8, 10, 12, 20 and 32 weeks of healing. The graphs include32 values quantified by Anonymous *et al.* [18] and those presented in this paper.


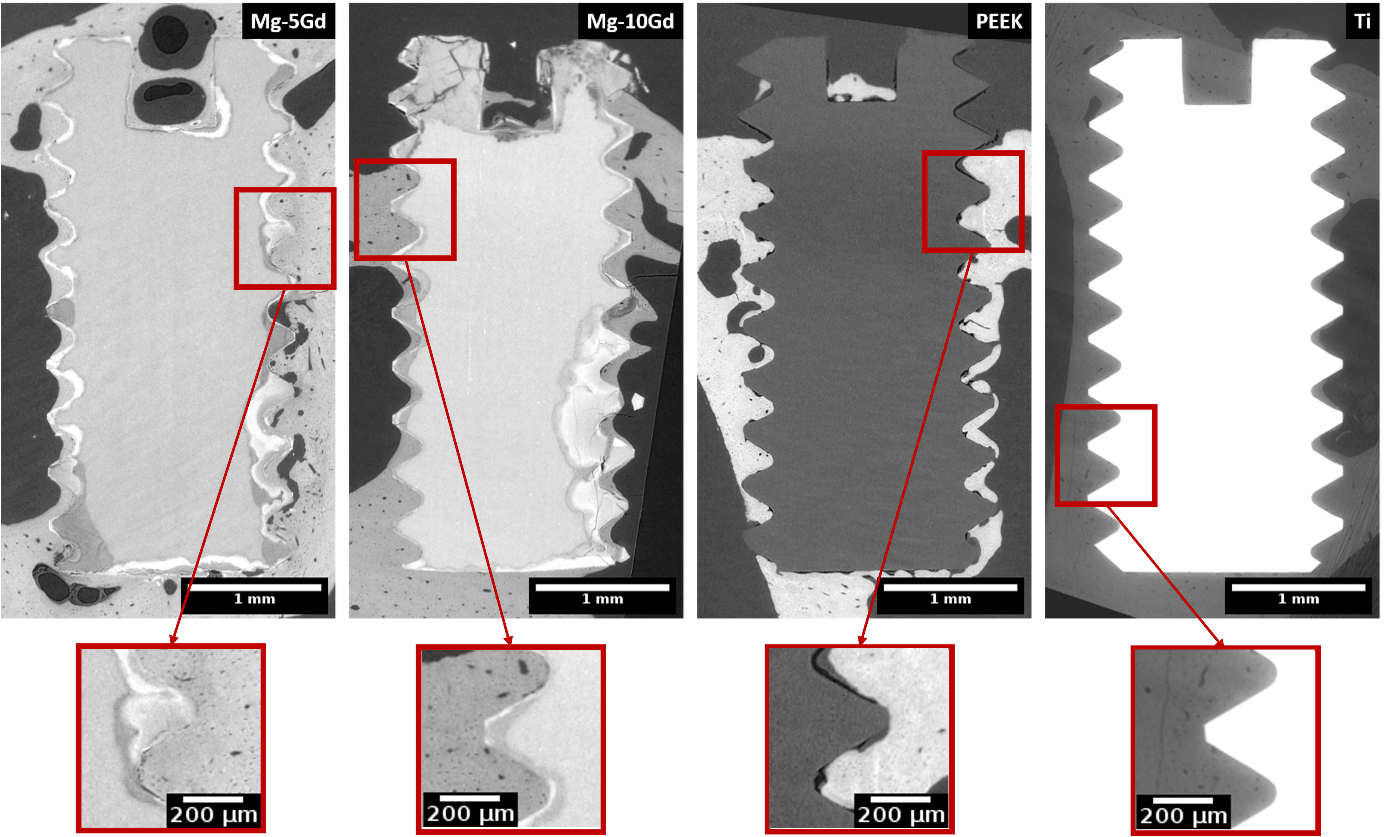


Figure 12: Longitudinal sections of *ex vivo* SRµCT scans of Mg-5Gd, Mg-10Gd, PEEK and Ti samples after 32 weeks post-implantation. The image contrast was adjusted for better visualization. Representative images of BIC parameter.

## DR [mm/year] BIC [%] BV/TV [%]

| **4 weeks** | Mg-5Gd | 0.63±0.16 | 25.0±7.7 | 23.6±3.9 |
| --- | --- | --- | --- | --- |
|  | Mg-10Gd | 0.62±0.24 | 36.0±13.0 | 25.1±8.5 |
|  | PEEK | - | 7.5±3.8 | 36.5±7.7 |
|  | Ti | - | 37.7±15.0 | 48.8±10.4 |
| **8 weeks** | Mg-5Gd | 0.37±0.05 | 49.2±6.3 | 36.5±4.3 |
|  | Mg-10Gd | 0.39±0.08 | 48.0±7.8 | 34.9±5.0 |
|  | PEEK | - | 19.5±5.5 | 42.8±5.8 |
|  | Ti | - | 57.3±11.5 | 51.3±4.6 |
| **10 weeks** | Mg-5Gd | 0.28±0.07 | 60.6±7.0 | 40.7±8.0 |
|  | Mg-10Gd | 0.32±0.09 | 60.0±17.3 | 33.3±9.9 |
|  | PEEK | - | 30.0±15.6 | 40.5±4.6 |
|  | Ti | - | 69.5±8.6 | 40.8±7.9 |
| **12 weeks** | Mg-5Gd | 0.34±0.05 | 53.1±10.4 | 45.2±4.3 |
|  | Mg-10Gd | 0.26±0.07 | 54.3±6.5 | 47.6±6.2 |
|  | PEEK | - | 27.2±5.8 | 49.4±5.7 |
|  | Ti | - | 51.1±14.9 | 49.1±7.7 |
| **20 weeks** | Mg-5Gd | 0.14±0.02 | 71.6±11.7 | 42.4±7.0 |
|  | Mg-10Gd | 0.16±0.01 | 75.9±3.0 | 44.1±3.3 |
|  | PEEK | - | 44.8±13.7 | 46.6±9.5 |
|  | Ti | - | 79.4±3.8 | 43.2±6.2 |
| **32 weeks** | Mg-5Gd | 0.08±0.01 | 80.8±6.0 | 55.4±7.2 |
|  | Mg-10Gd | 0.08±0.01 | 77.1±3.0 | 45.8±4.8 |
|  | PEEK | - | 53.5±13.7 | 49.6±4.8 |
|  | Ti | - | 82.9±11.5 | 55.7±11.7 |

Table 5: Summary of mean values with standard deviation of DR, BIC and BV/TV parameters describing Mg-5Gd, Mg-10Gd, PEEK and Ti explants after 4, 8, 10, 12, 20 and 32 weeks of healing. Table include values quantified by Anonymous et. al [18] (4, 8 and 12 weeks) and the ones analysed in this paper (10, 20 and 32 weeks).


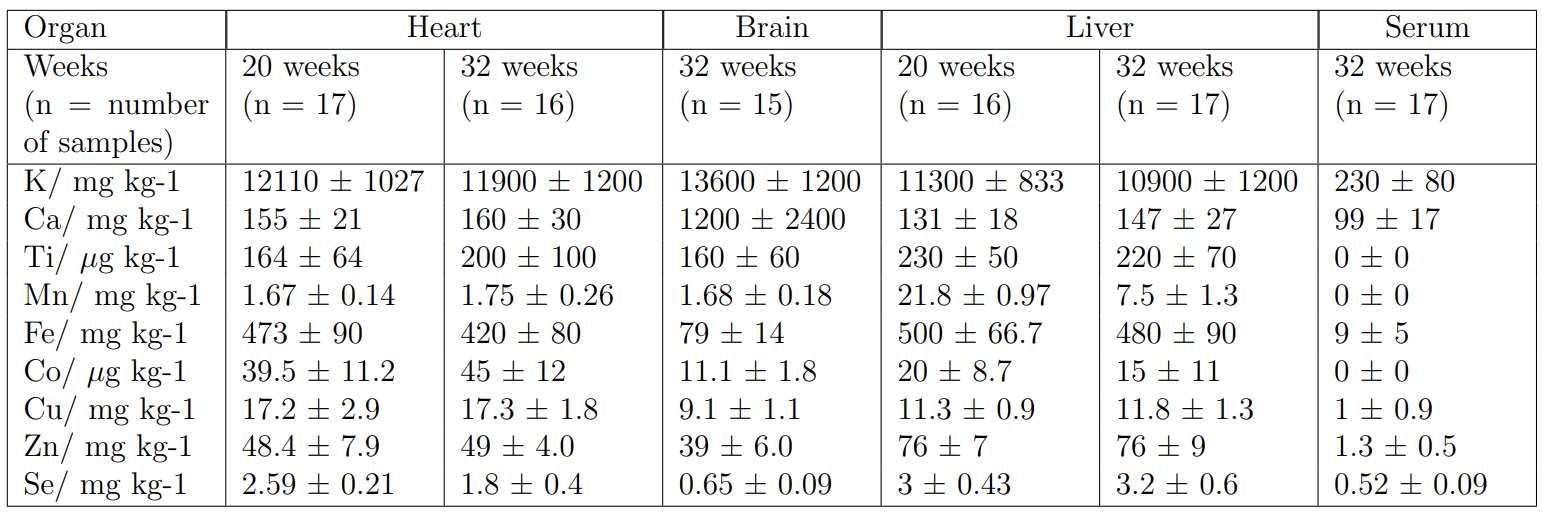


Table 6: Essential elements in selected organs and serum.


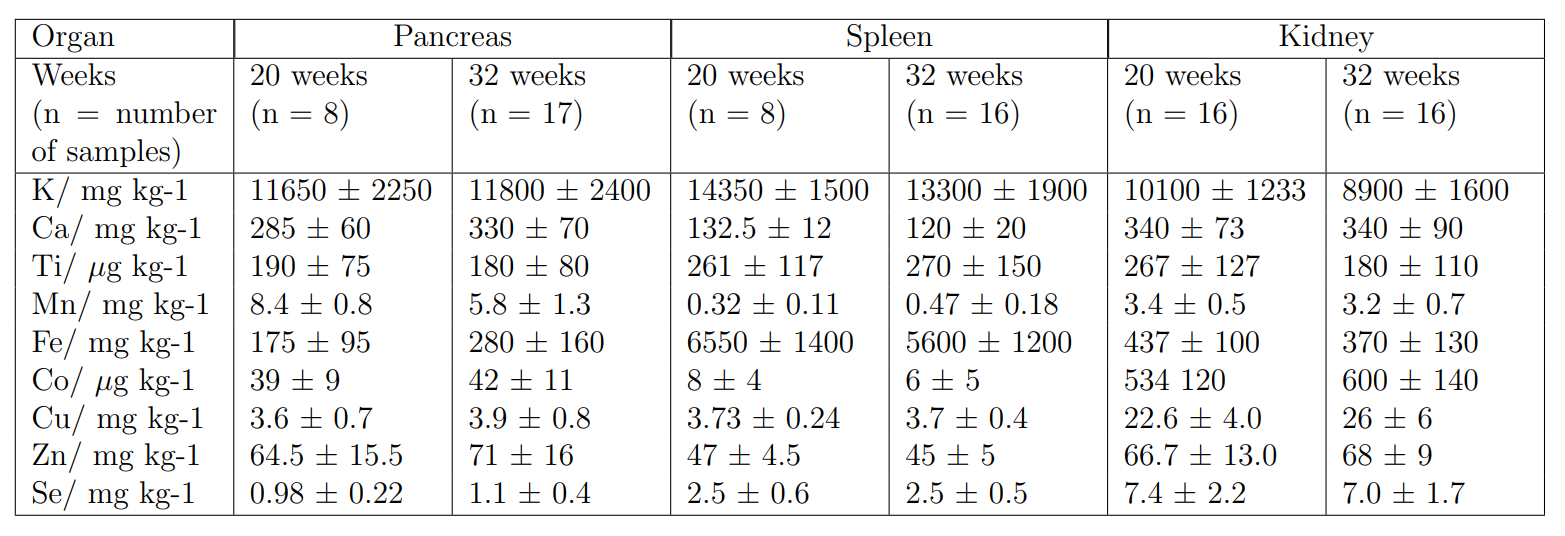


Table 7: Determination of trace and essential elements in selected organs.


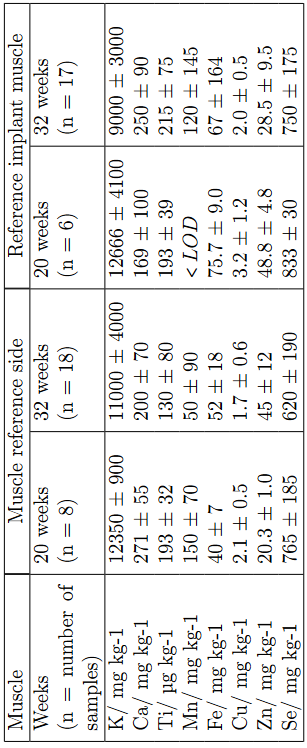


Table 8: Determination of trace and essential elements in muscles obtained from reference sides; LOD Limit of determination.


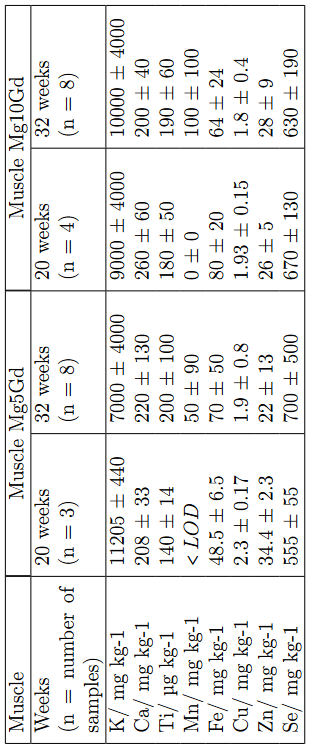


Table 9: Determination of trace and essential elements in the muscle in vicinity to the implant side; LOD-Limit of determination.


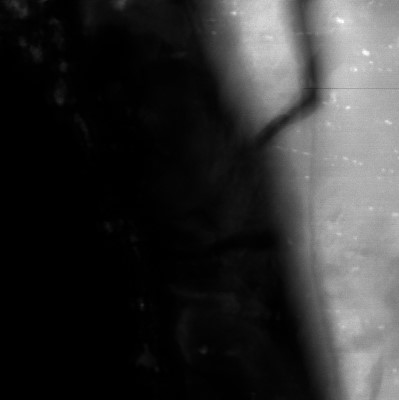

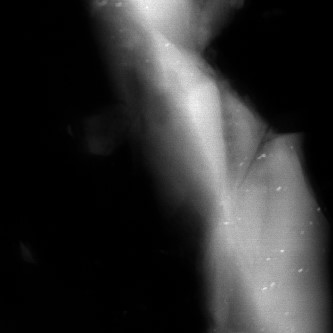

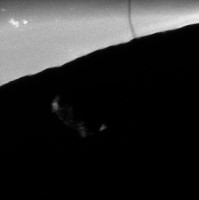

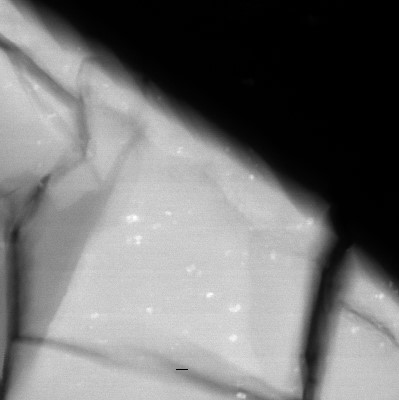


Figure 13: Gd distribution maps of the degradation layer formed on the surface of Mg10Gd implants after 4 (a), 10 (b), 20 (c), and 32 (d) months after the implantation. All the maps have a size of 100 x 100 um^2^.


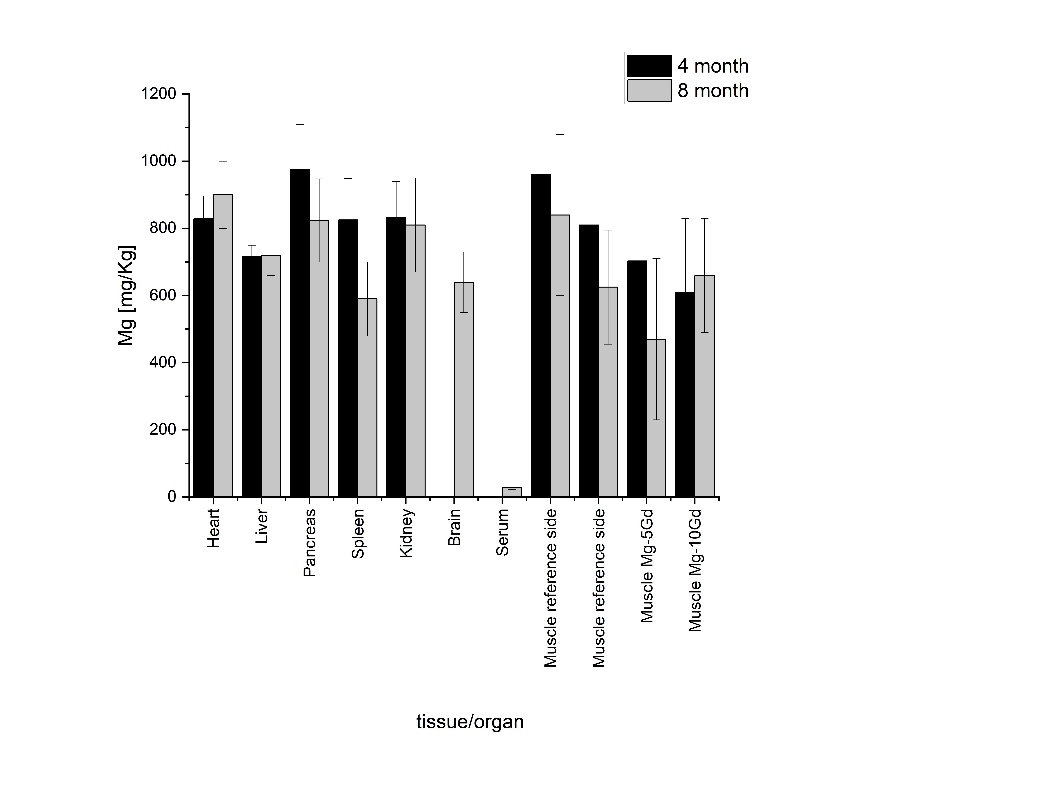

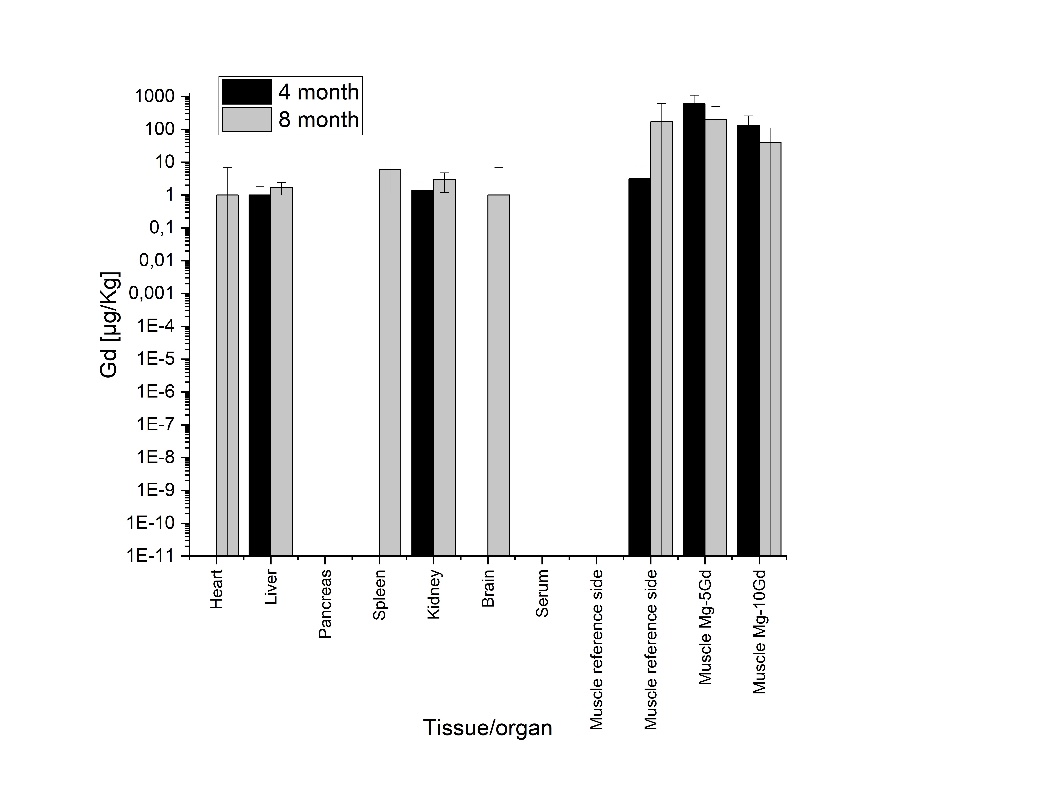


Figure 14: Mg and Gd distribution in selected organs determined using ICP-MS/MS.

as also shown in table 4.


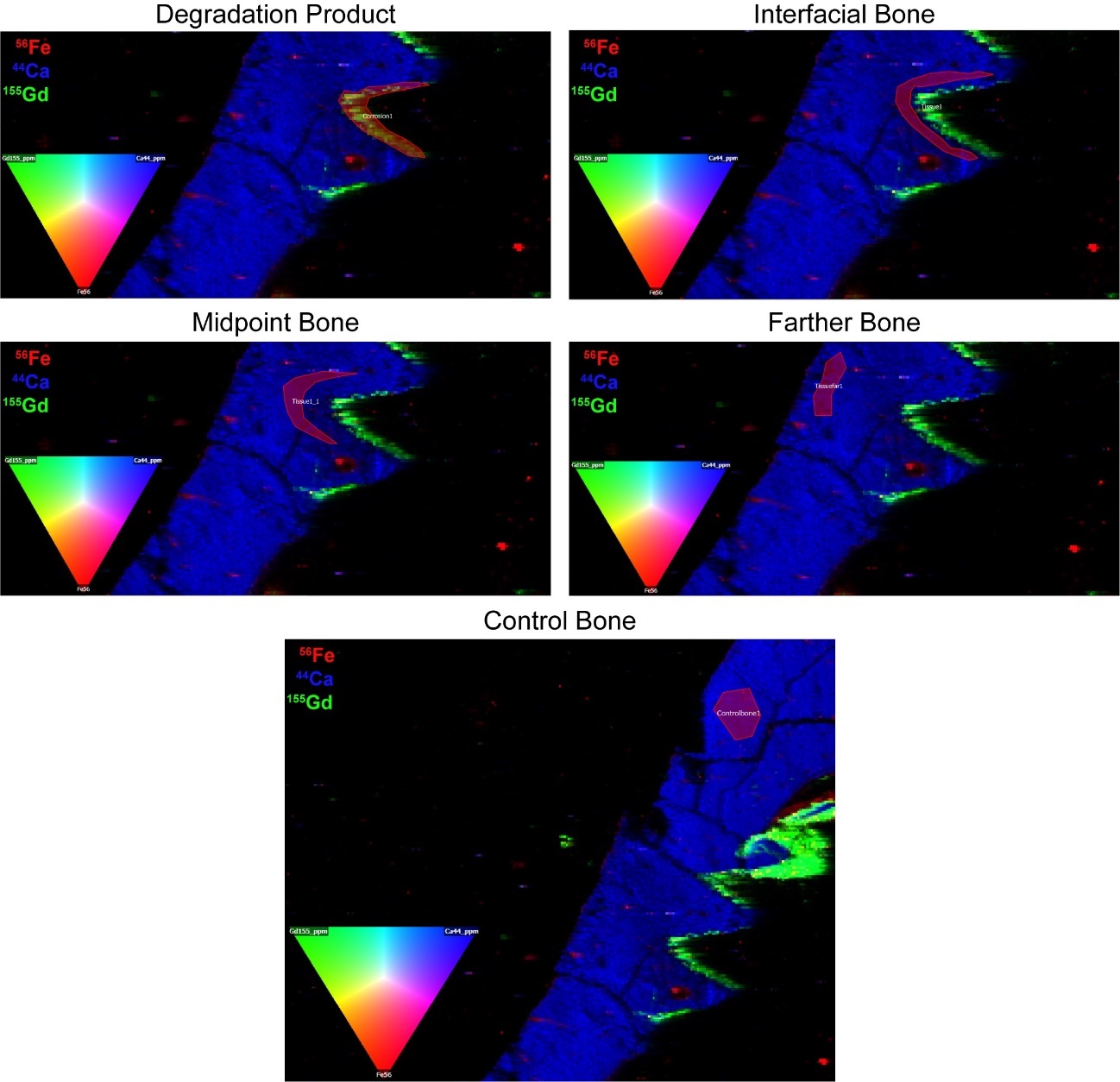


Figure 15: Demonstration of region selection for bone implants, using screw thread corrosion product remnants to inform material- tissue barriers. A) corrosion product, B) Interfacial tissue C)-D) a region progression showing tissue further away from the interface and E), control bone tissue away from ROI


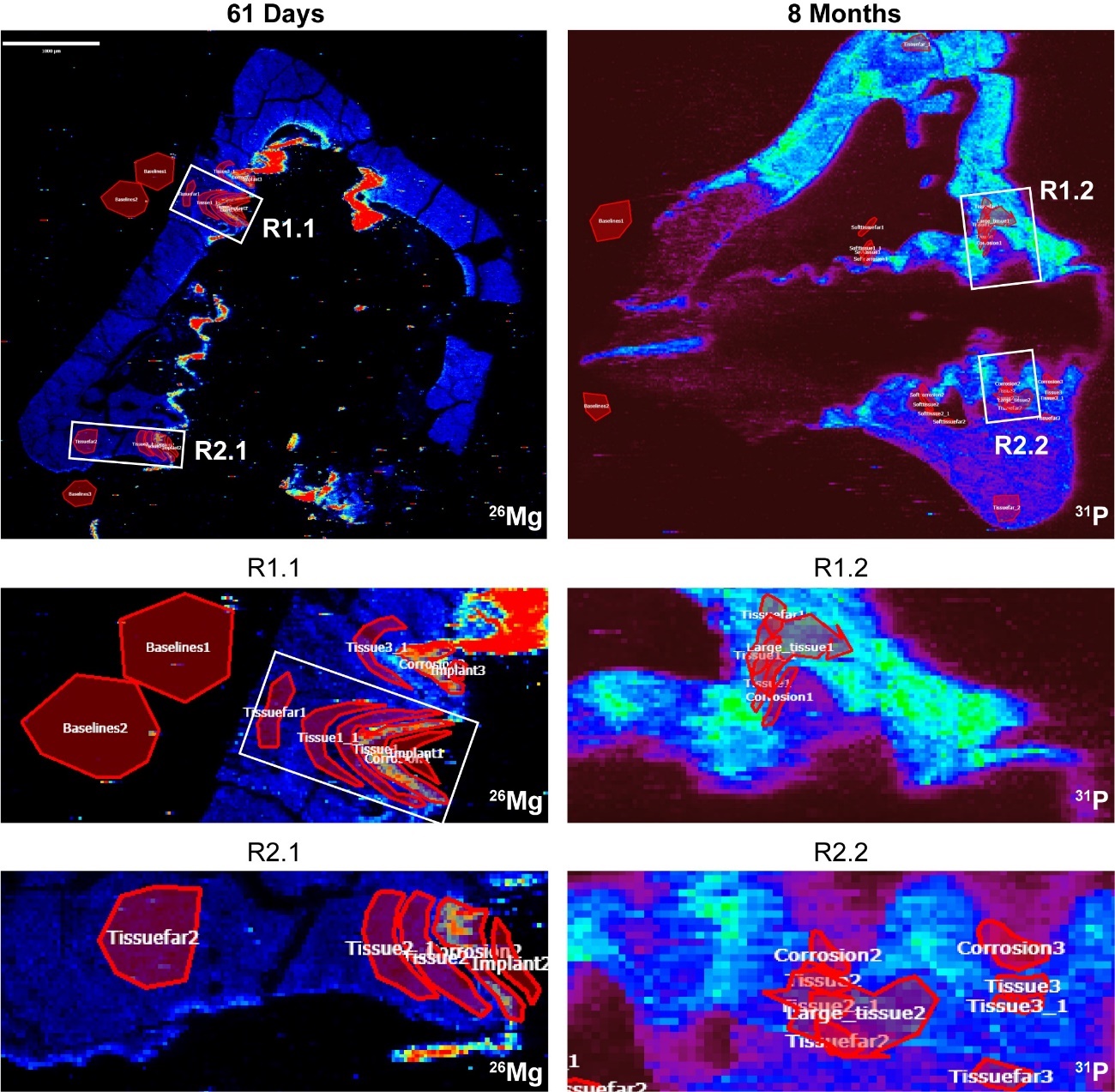


Figure 16: Precise region assignment for the Mg-10Gd material on LITM scans, which correspond to regions outlined.


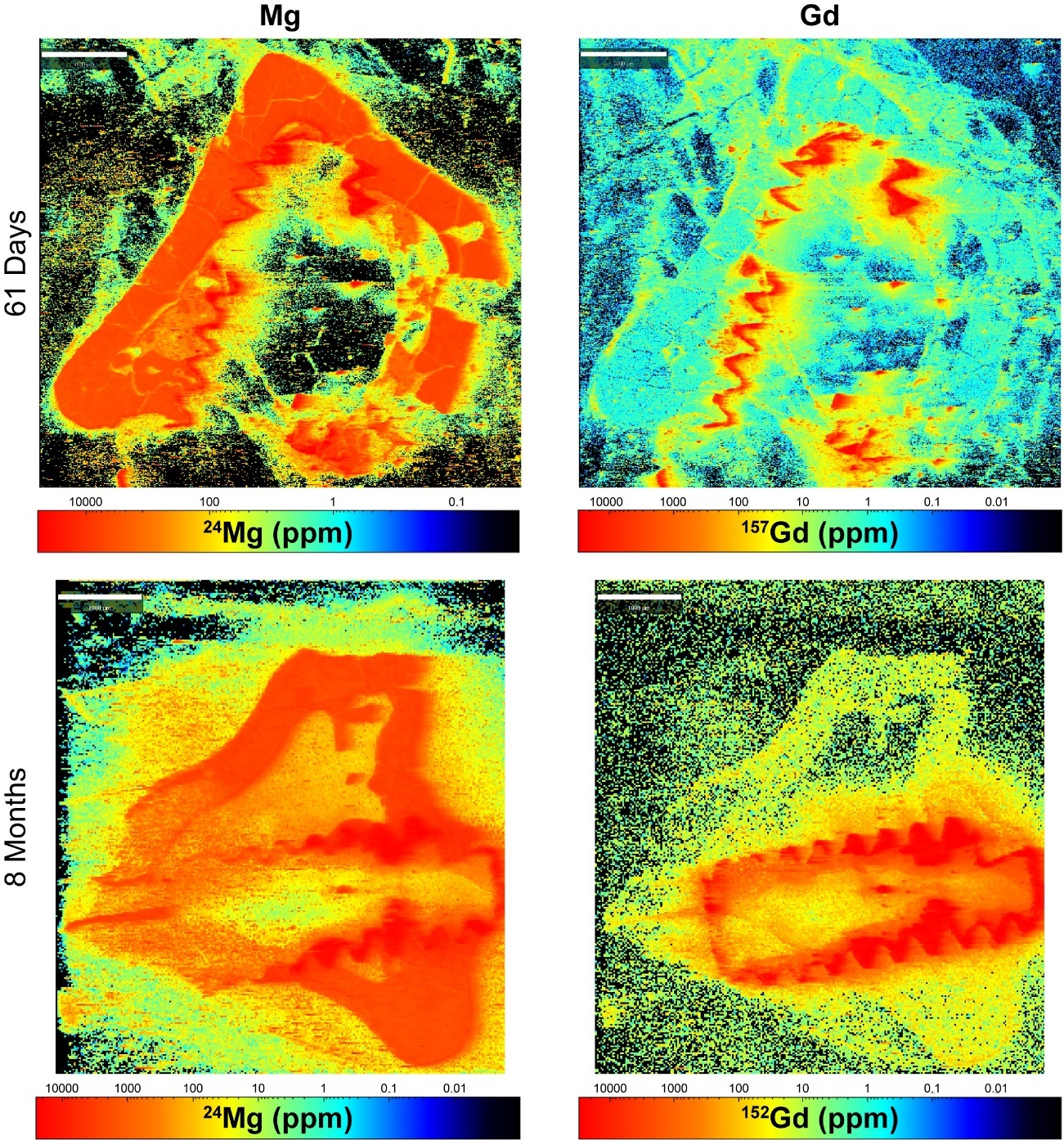


Figure 17: Logarithmic scaling of pixels of Mg-10Gd at each timepoint for M and Gd. Arrows in 8-month row demonstrate changes in the distribution of high value pixels at similar interfacial regions for Mg vs. Gd


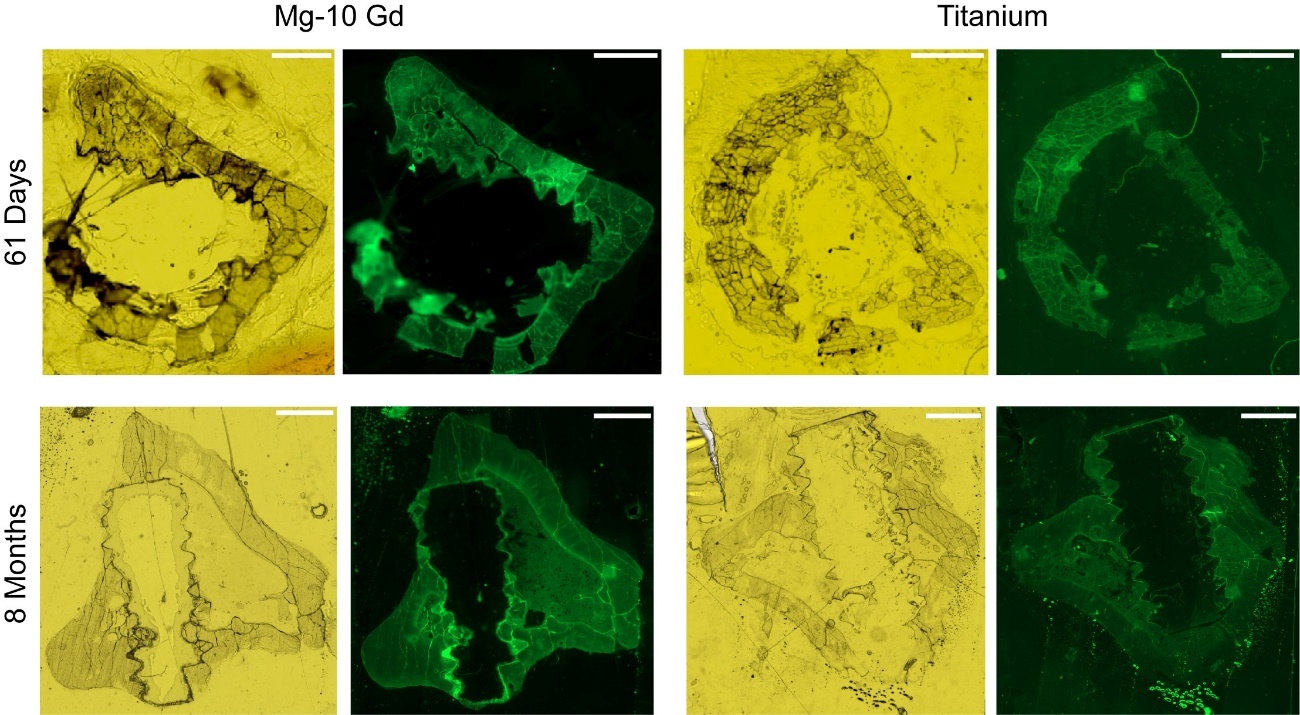


Figure 18: Logarithmic scaling of pixels of Mg-10Gd at each timepoint for M and Gd. Arrows in 8-month row demonstrate changes in the distribution of high value pixels at similar interfacial regions for Mg vs. Gd

**Section SI 1: Gelatin standard preparation and measurement for the calibration of the TOF -MS data**

***Custom Gelatin Standard Preparation***

We prepared two sets of homogeneous 10% (m/v) gelatin calibration standards. Porcine gelatin with 300 bloom strength was used for all gelatin preparations.

**Stock 1** contained high concentrations of Mg, Ca, and Gd which we prepared in 7.7 mL of ultrapure, deionized water (18.2 MΩ·cm @ 25 °C) @ 65 °C using the components below:

**Table 1.** Stock 1 gelatin standard composition

| **Compound** | **Company** | **Cat #** | **Mass (mg)** |
| --- | --- | --- | --- |
| Calcium Chloride Dihydrate | Sigma Aldrich | 21097-250G | 1001.83 |
| Magnesium Chloride | Sigma Aldrich | M8266-100G | 101.17 |
| Gadolinium Acetate Hydrate | Sigma Aldrich | 325678-25G | 189.69 |
| Gelatin | Sigma Aldrich | 1010.03 | 1010.03 |

Stock 1 standard was then mixed gently using metal free plastic spatulas and degassed in an ultrasonic water bath set at 65 °C to remove any air bubbles from mixing. The **Stock 1** standard was then serially diluted into fresh 10% (w/v) gelatin in ultrapure deionized water to make 100, 50, 25, 12.5, 6.25, and 3.125 mg/g Ca.

**Stock 2** gelatin standards were made by diluting a 1000 µg/mL Ti individual element standard (in 2% v/v nitric acid/trace hydrofluoric acid) and a 1000 µg/mL multi-element standard containing Ca, K, Ba, B, Cr, Cu, In, Pb, Mn, Ag, Sr, Zn, Mg, Al, Bi, Cd, Co, Ga, Fe, Li, Ni, Na, Tl (IV-Stock-IV, in 5% v/v nitric acid) into 8 mL of ultrapure, deionized water (18.2 MΩ·cm @ 25 °C) @ 65 °C and adding ~ 1 g of gelatin (see components below):

**Table 2.** Stock 2 gelatin standard composition

| **Standard** | **Company** | **Cat #** | **Amount** |
| --- | --- | --- | --- |
| Titanium Standard | Inorganic Ventures | CGTI1-125ML | 500 µL |
| Multi-element IV-STOCK-4 | Inorganic Ventures | IV-STOCK-4 | 500 µL |
| Gelatin | Sigma Aldrich | 1010.03 | 1012.43 |

Stock 2 standard was then mixed gently using metal free plastic spatulas and degassed in an ultrasonic water bath set at 65 °C to remove any air bubbles from mixing. The **Stock 2** standard was then serially diluted into fresh 10% (w/v) gelatin in ultrapure deionized water to make 50, 25, 12.5, 6.25, and 3.125 ng/g element.

***Sample Preparation for Inductively Coupled Plasma (ICP) Analysis***

All standards, blanks, and gelatin samples were prepared using trace metal grade nitric acid (70%, Fisher chemical, Cat# A509P212), ultrapure water (18.2 MΩ·cm @ 25 °C), and metal free polypropylene conical tubes (15 and 50 mL, Labcon, Petaluma, CA, USA). After preparing liquid gelatin standards and blanks, 50 µL of each standard was added to a pre-weighed 15 mL metal free conical tube and capped as to avoid evaporation of the heated liquid. After allowing the gelatin to cool (at least 4 hours at room temperature), 300 µL of distilled trace metal grade 70% nitric acid was added to each tube and digested at 70 °C for 4 hours. Following digestion, 9.7 mL of ultrapure deionized H_2_O was added, and the sample was weighed.

***ICP-Optical Emission Spectroscopy (OES) analysis of Stock 1 Gelatin Standards***

Stock 1 gelatin standards were analyzed using an Agilent 5800 ICP-OES (Agilent, Santa Clara, CA, USA) equipped with the Agilent SPS 4 Autosampler, quartz cyclonic spray chamber, micromist nebulizer, and AVS 6/7 valve introduction system. Daily instrument performance is validated via a detector calibration and multiwavelength calibration in axial and radial mode using the manufacturer’s multi-element calibration standard containing 50 mg/L Al, As, Ba, Cd, Co, Cr, Cu, Mn, Mo, Ni, Pb, Se, Sr, Zn and 500 mg/L K in 5% nitric acid. ICP-MS standards were prepared from a stock solution of IV-Stock-3 multi-element standard (Ca, Mg, K, Na) as well as Gd individual standard (Inorganic Ventures, Christiansburg, VA, USA) that were diluted with 3% (v/v) trace nitric acid in ultrapure deionized water to a final element concentration of 100, 10, 1, 0.1, and 0 (blank) µg/g standard. Internal standardization was accomplished inline using the AVS 6/7 valve and a 1 µg/g internal standard solution in 3% (v/v) trace nitric acid in ultrapure water consisting of Y (Inorganic Ventures, Christiansburg, VA, USA). The emission lines selected (in both axial and radial mode) for analysis were Ca (422.673 nm), Gd (342.246 nm), Mg (279.800 nm), and Y (371.029 nm) used for internal standardization (see **table 3** for concentrations).

*
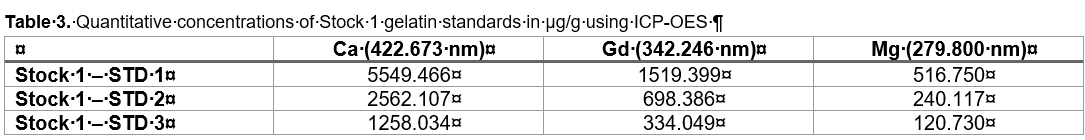
*

***ICP Triple Quadrupole Mass Spectrometry (ICP-QQQ-MS) Analysis of Stock 2 Gelatin Standards***

Stock 2 gelatin standards were analyzed using an Agilent 8900 Triple Quadrupole ICP-MS (Agilent, Santa Clara, CA, USA) equipped with the Agilent SPS 4 Autosampler, integrate sample introduction system (ISiS), x-lens, and micromist nebulizer. Daily tuning of the instrument was accomplished using the manufacturer supplied tuning solution containing Li, Co, Y, Ce, and Tl. Global tune optimization was based on optimizing intensities for ^7^Li, ^89^Y, and ^205^Tl while minimizing oxides (^140^Ce^16^O/^140^Ce < 1.5%) and doubly charged species (^140^Ce++/^140^Ce+ < 2%). Following global instrument tuning, gas mode tuning in He KED and O_2_ mode was accomplished using the same manufacturer supplied tuning solution. In KED mode (using 100% UHP He, Airgas), intensities for ^59^Co, ^89^Y, and ^205^Tl were maximized while minimizing oxides (^140^Ce^16^O/^140^Ce < 0.5%) and doubly charged species (^140^Ce++/^140^Ce+ < 1.5%) with short term RSDs < 3.5%. In O_2_ mode (using 100% UHP O_2_, Airgas) intensities for ^59^Co, ^89^Y, and ^205^Tl with short term RSDs < 3.5%. ICP-MS standards were prepared from a stock solution of IV-Stock-4 multi-element standard (Ca, K, Ba, B, Cr, Cu, In, Pb, Mn, Ag, Sr, Zn, Mg, Al, Bi, Cd, Co, Ga, Fe, Li, Ni, Na, Tl) as well as Ti and Gd individual standards (Inorganic Ventures, Christiansburg, VA, USA) that were diluted with 3% (v/v) trace nitric acid in ultrapure deionized water to a final element concentration of 10000 (1000 Ti), 1000 (100 Ti), 100 (10 Ti), 10 (1 Ti), 1 (0.1 Ti), 0.1 (0.01 Ti), and 0 (blank) ng/g standard. Internal standardization was accomplished inline using the ISIS valve and a 200 ng/g internal standard solution in 3% (v/v) trace nitric acid in ultrapure water consisting of Bi, In, ^6^Li, Sc, Tb, and Y (IV-ICPMS-71D, Inorganic Ventures, Christiansburg, VA, USA). The isotopes selected for analysis were ^23^Na, ^39^K, ^47^Ti, ^52^Cr, ^55^Mn, ^57^Fe, ^59^Co, ^60^Ni, ^65^Cu, ^66^Zn, ^111^Cd, and ^157^Gd with ^6^Li, ^45^Sc, and ^89^Y used for internal standardization.

**Table 4.** Quantitative concentrations of Stock 2 gelatin standards in µg/g using ICP-QQQ-MS

**
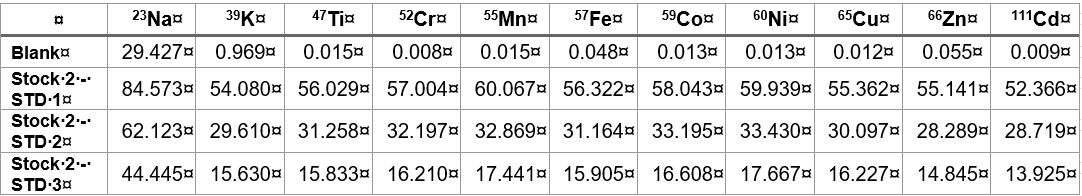
**

***Gelatin Standard Sectioning Protocol***

After making 6 Stock 1 and 6 Stock 2 gelatin standards (including one blank gelatin solution), the gelatin was prepared for cryosectioning using a Leica CM 3050 S (Leica Biosystems, Deer Park, IL, USA) cryostat. After the gelatin is dissolved and the solution is still at 55 °C, we pipetted approximately 300 µL of warmed gelatin standard onto a precooled chuck inside the cryostat (-20 °C). We allowed the gelatin to freeze for no less than 4 minutes at -20 °C. Once the gelatin standards were completely frozen, we sectioned samples with a -21 °C chamber temperature and -20 °C objective temperature (temperatures were adjusted slightly depending on the quality of the sections and whether the section stuck to the blade or anti-roll plate) and a section thickness of 10 µm to match the section thickness of the tissue. Gelatin sections were then transferred to pre-cleaned charged microscope slides (Superfrost plus, thermos scientific) or Kapton tape attached to pre-cleaned charged microscope slides and kept at -20 °C until laser ablation.

***LA-ICP-TOF-MS instrument setup and sample parameters***

Glass slides were loaded into a Bioimage 266 nm laser ablation system (Elemental Scientific Lasers, Bozeman, MT, USA) which is equipped with an ultra-fast low dispersion TwoVol3 ablation chamber and a dual concentric injector (DCI3) and is coupled to an icpTOF S2 (TOFWERK AG, Thun, Switzerland) ICP-TOF-MS. Daily tuning of the LA-ICP-TOF-MS settings was performed using NIST SRM612 glass certified reference material (National Institute for Standards and Technology, Gaithersburg, MD, USA). Optimization for torch alignment, lens voltages, and nebulizer gas flow was based on high intensities for ^140^Ce and ^55^Mn while maintaining low oxide formation based on the ^232^Th^16^O+/^232^Th+ ratio (< 0.5). A list of instrument parameters for LA-ICP-TOF-MS is summarized in Table S1.

In short, 10 µm thick gelatin standards (3 Stock 1 standards, 3 Stock 2 standards, and a blank) were placed directly on charged slides or Kapton tape which was then placed sticky side up on charged slides. These 2 slides were loaded with a sample slide on the LA sample holder and loaded into the LA system. The system is purged for 5 minutes and patterns are selected on the standards and samples of interest. For gelatin standards, 9 lines going across each standard and the blank were drawn using the same laser parameters as the samples (see table). Specifically, 10 or 20 µm circular laser spot sizes at 80 or 100% laser power and 100 Hz repetition rate were used with an interline distance 4 times greater than the spot size. This allowed for clean ablation of individual lines without overlap. For the sample, reference points were made around the sample and the aforementioned laser parameters were used with an interline distance equal to the laser spot with no overlap to sample the entire tissue.

***Data Acquisition and Analysis of LA-ICP-TOF-MS Data***

Data was recorded using TofPilot 1.3.4.0 (TOFWERK AG, Thun, Switzerland). The LA-ICP-TOFMS data were saved in the open-source hierarchical data format (HDF5). Post-acquisition data processing was performed with Tofware v3.2.0, which is a TOFWERK data analysis package and used as an add-on on IgorPro (Wavemetric Inc., OR, USA). The data processing comprised the following steps: (1) drift correction of the mass peak position in the spectra over time via time-dependent mass calibration, (2) determining the peak shape, and (3) fitting and subtracting the mass spectral baseline. The data was further processed with Iolite version 4.8.6 (Elemental Scientific Lasers, Bozeman, MT, USA). For calibration, signal responses for each ablation line per gelatin standard were fit to a linear regression and spline auto smoothed. Calibration curves are then generated and using 3D trace elements inside of DRS in Iolite, we can convert integrated counts per second to µg/g (using the previous calculations for the gelatin standards via ICP). Representative calibration curves are shown below.

**Table S10.** ICP-TOF-MS and laser ablation parameters for LA-ICP-TOF-MS analysis of tissues

| *ICP-MS Parameters (Tofwerk S2)* | | | |
| --- | --- | --- | --- |
| Parameter | **Unit** | **Value** |  |
| RF Power | W | 1550 |  |
| Sampling Depth | mm | 4.9 |  |
| Cone Material | Nickel | - |  |
| Cone Insert (STD) | mm | 3.5 |  |
| Plasma Gas Flow | L/min | 14.0 |  |
| Auxillary Gas Flow | L/min | 8.0 |  |
| Nebulizer Gas Flow | L/min | 0.9 - 1.0 |  |
| Measurement Mode | CCT Mode | - |  |
| CCT Gas Flow (100% He) | mL/min | 5 |  |
| CCT Focus lens | V | -19.5 |  |
| CCT Entry Lens | V | -180 |  |
| CCT Mass | V | 150 |  |
| CCT Bias | V | -27 |  |
| CCT Exit Lens | V | -200 |  |
| *Time-of-Flight Parameters (Tofwerk S2)* | | | |
| Parameter | **Unit** | **Value** |  |
| m/z range | amu | 14 - 256 |  |
| Resolution | m/Δm | 1000 |  |
| ODG Settings | ms | 20 |  |
| Notch Bias | V | -80 |  |
| Notch (40 amu) | V | 1.5 |  |
| Notch (156 amu) | V | 0.8 |  |
| Notch (28 amu) | V | 1.5 |  |
| Notch (15.8 amu) | V | 3 |  |
| *Laser Ablation Parameters (ESL Bioimage 266 nm)* | | | |
| Parameter | **Unit** | **Value** | **Value** |
| **Spot Size** | µm | 10 | 20 |
| **Interline Distance (y)** | µm | 10 | 20 |
| **Overlap (x)** | µm | 0 | 0 |
| **Repetition Rate** | Hz | 100 | 100 |
| **Laser Power** | % | 80 | 100 |
| **Laser Fluence** | J/cm^2^ |  |  |
| **Sample Energy** | mJ |  |  |
| **Imaging Cup Flow Rate (He)** | mL/min | 300 |  |
| **Imaging Chamber Flow Rate (He)** | mL/min | 300 |  |
| **PEEK Tubing I.D.** | mm | 1 |  |

**Supporting Figure 19.** Calibration curves for 10Gd_21TL_61d


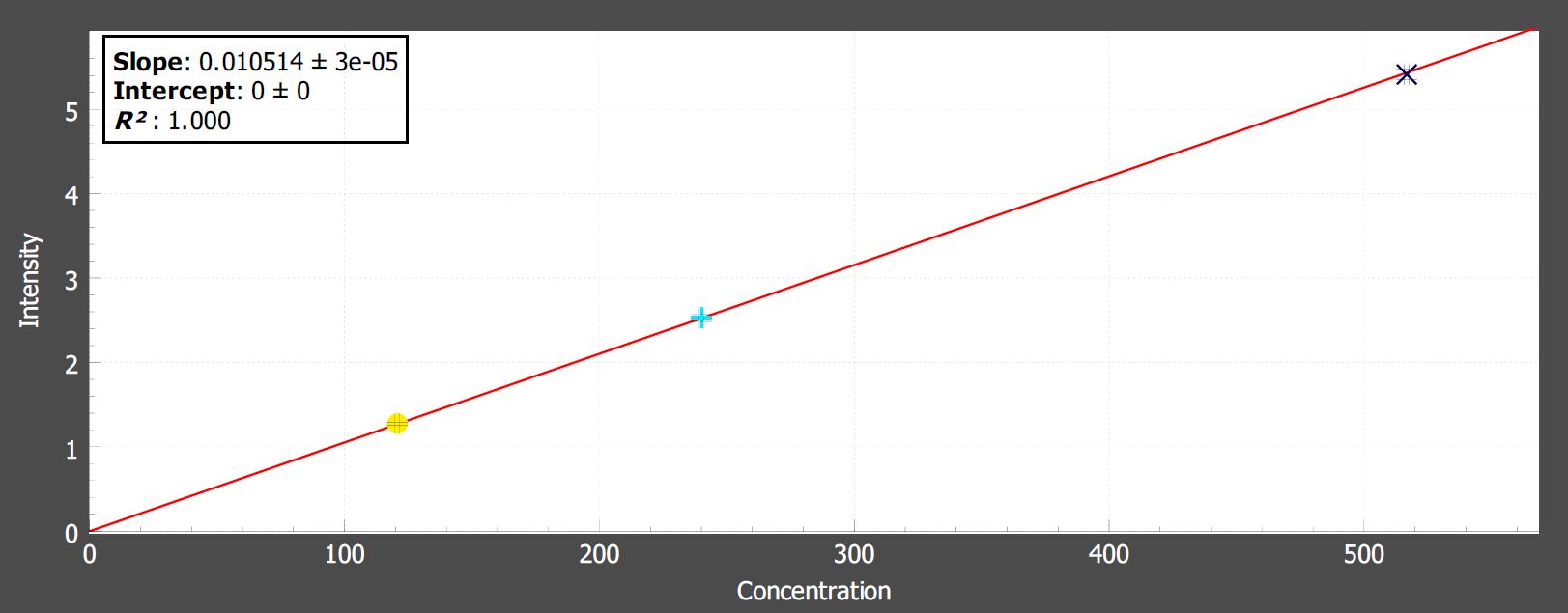


**^24^Mg**


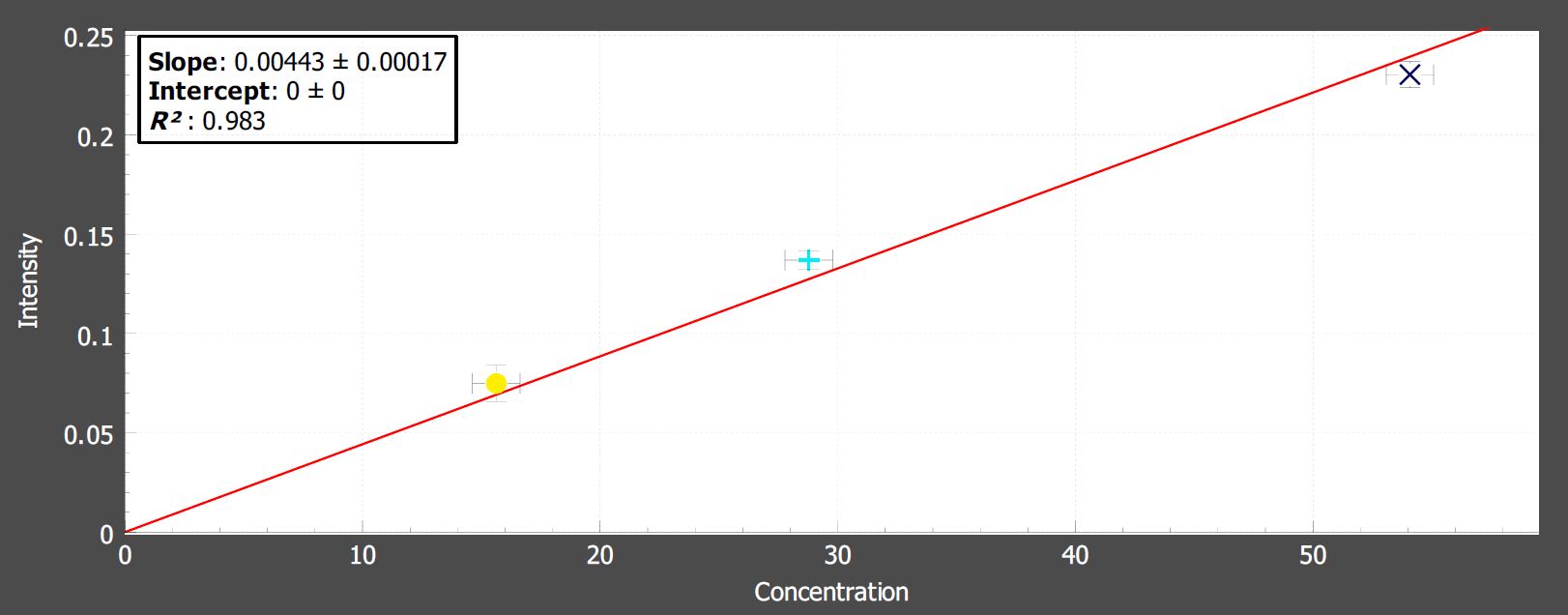


**^39^K**


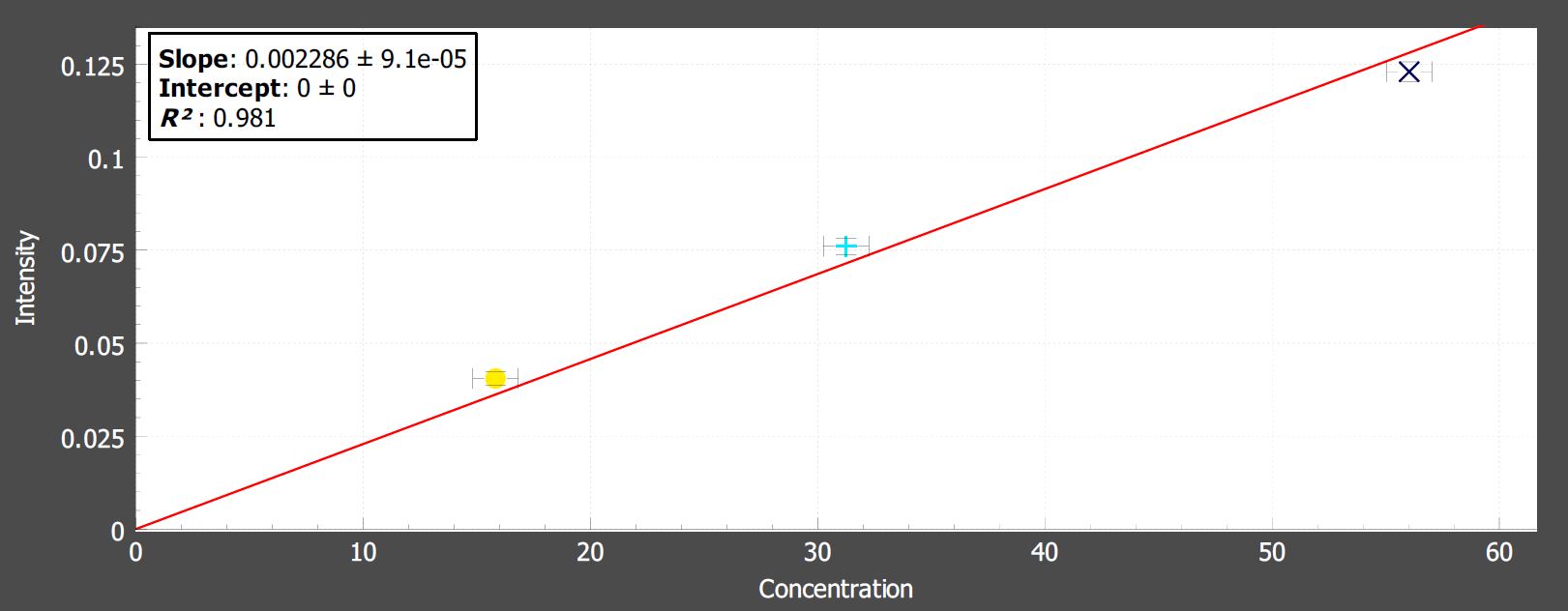


**^47^Ti**


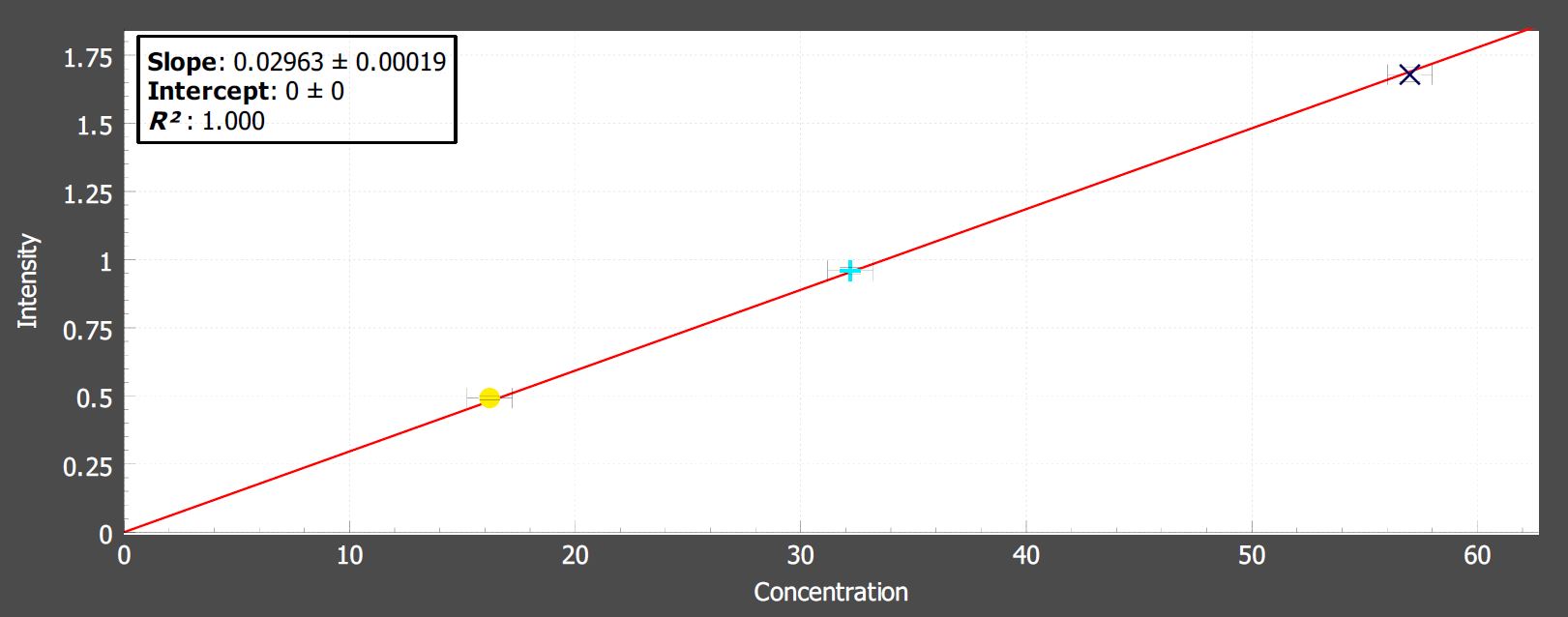


**^52^Cr**


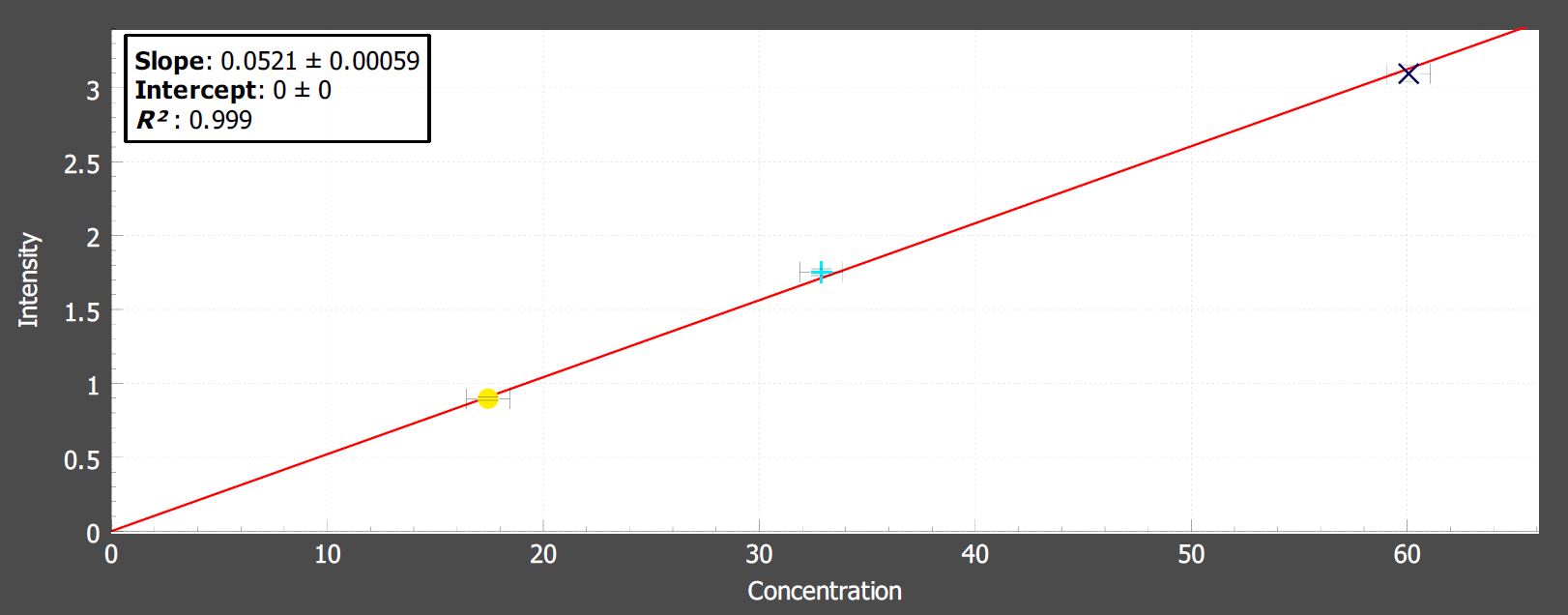


**^55^Mn**


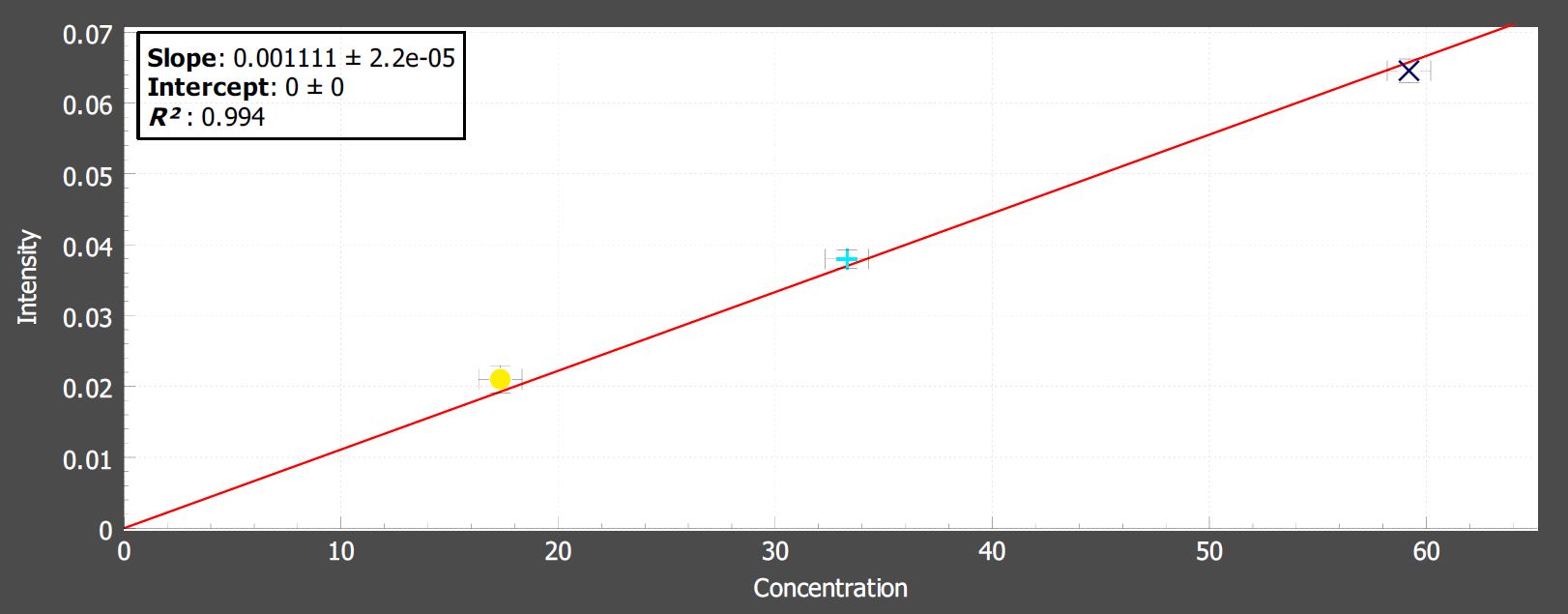


**^57^Fe**


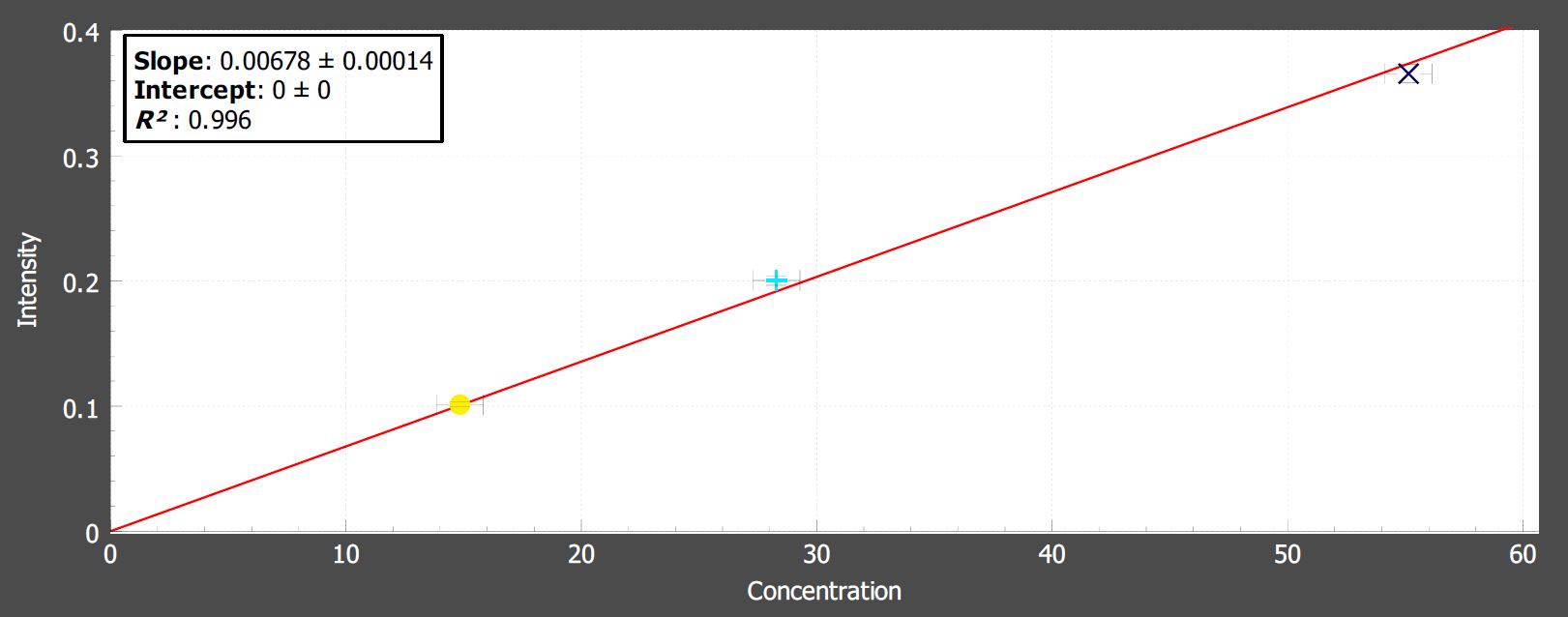


**^66^Zn**


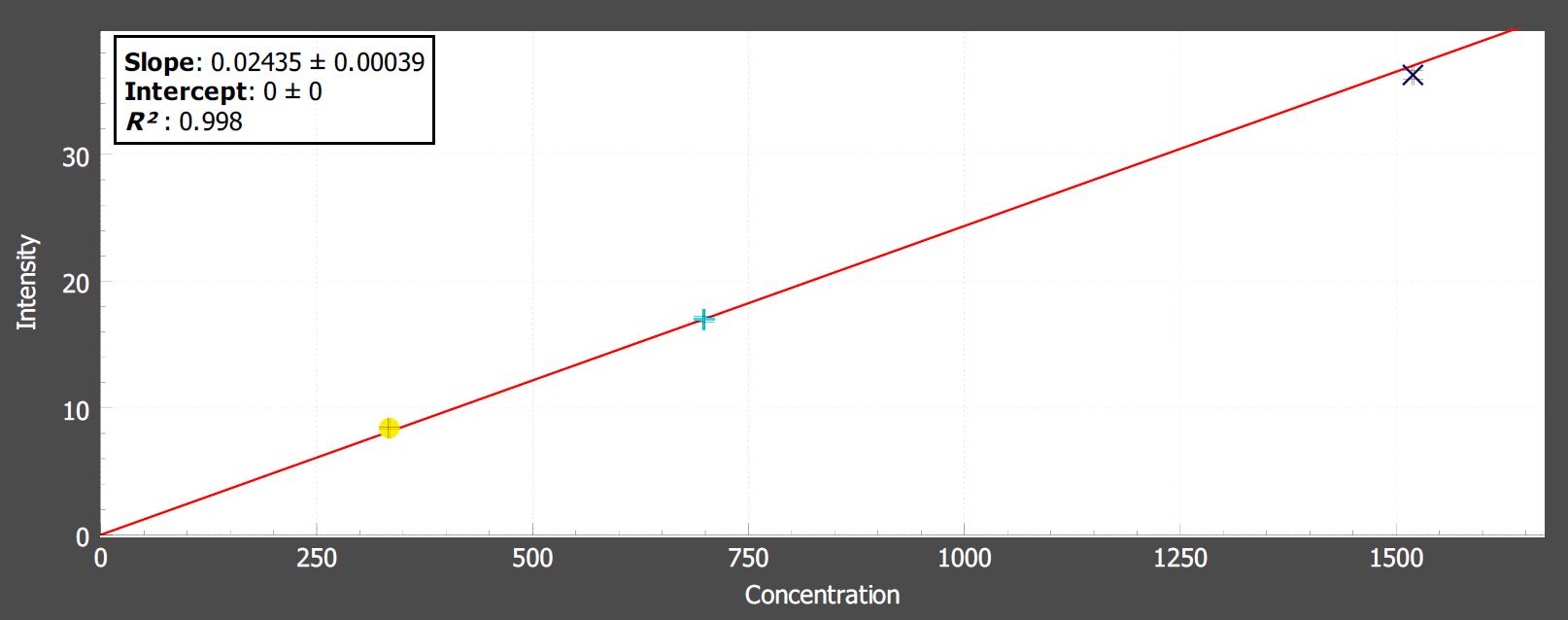


**^157^Gd**


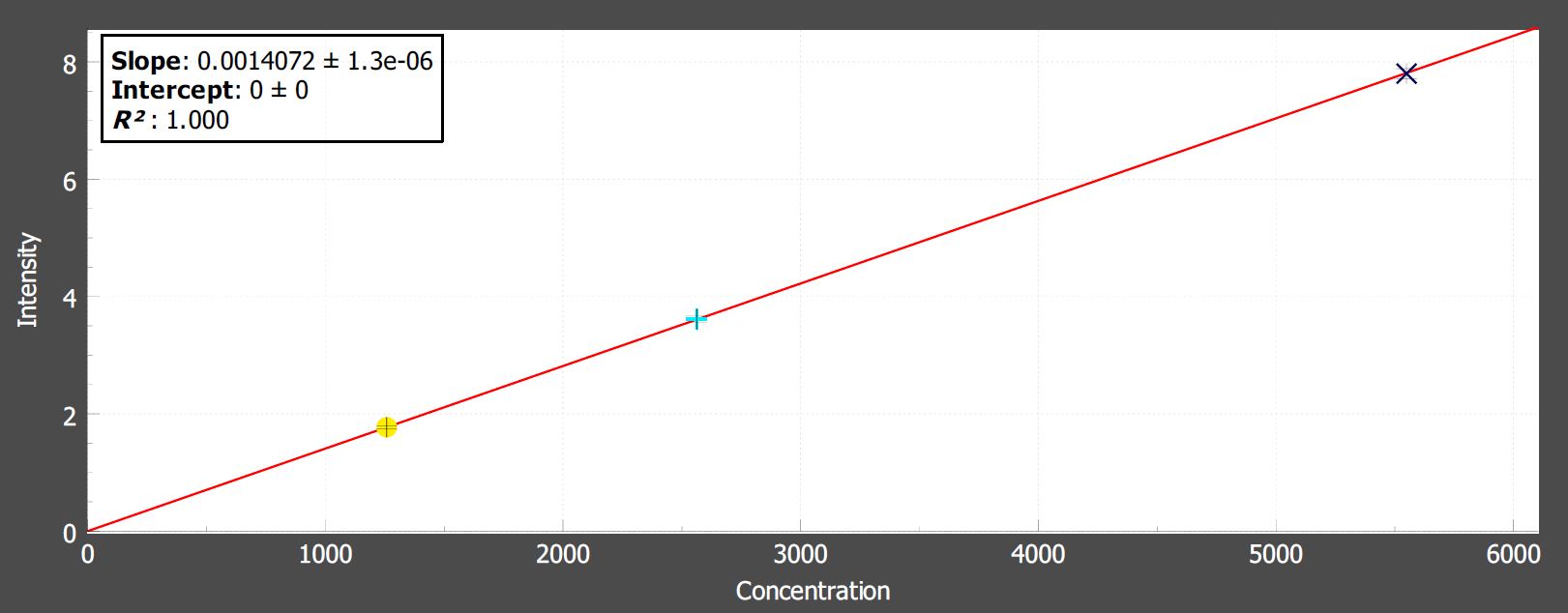


**^44^Ca**
